# High-throughput RNA sequencing of paraformaldehyde-fixed single cells

**DOI:** 10.1038/s41467-021-25871-2

**Published:** 2021-09-24

**Authors:** Hoang Van Phan, Michiel van Gent, Nir Drayman, Anindita Basu, Michaela U. Gack, Savaş Tay

**Affiliations:** 1 Pritzker School of Molecular Engineering, The University of Chicago, Chicago, IL USA; 2 Department of Microbiology, The University of Chicago, Chicago, IL USA; 3 Florida Research and Innovation Center, Cleveland Clinic, Port Saint Lucie, FL USA; 4 Department of Medicine, The University of Chicago, Chicago, IL USA

**Keywords:** RNA, Gene expression profiling, High-throughput screening, Viral infection

## Abstract

Single-cell transcriptomic studies that require intracellular protein staining, rare cell sorting, or inactivation of infectious pathogens are severely limited. This is because current high-throughput single-cell RNA sequencing methods are either incompatible with or necessitate laborious sample preprocessing for paraformaldehyde treatment, a common tissue and cell fixation and preservation technique. Here we present FD-seq (Fixed Droplet RNA sequencing), a high-throughput method for droplet-based RNA sequencing of paraformaldehyde-fixed, permeabilized and sorted single cells. We show that FD-seq preserves the RNA integrity and relative gene expression levels after fixation and permeabilization. Furthermore, FD-seq can detect a higher number of genes and transcripts than methanol fixation. We first apply FD-seq to analyze a rare subpopulation of cells supporting lytic reactivation of the human tumor virus KSHV, and identify *TMEM119* as a potential host factor that mediates viral reactivation. Second, we find that infection with the human betacoronavirus OC43 leads to upregulation of pro-inflammatory pathways in cells that are exposed to the virus but fail to express high levels of viral genes. FD-seq thus enables integrating phenotypic with transcriptomic information in rare cell subpopulations, and preserving and inactivating pathogenic samples.

## Abstract (web summary)

Current high-throughput single-cell transcriptomic methods are incompatible with paraformaldehyde, a common cell fixation technique. Here the authors present FD-seq, a method for droplet-based RNA sequencing of paraformaldehyde-fixed, stained and sorted single cells.

## Introduction

Single-cell RNA sequencing (scRNA-seq) has found many important biological applications, from the discovery of new cell types^1^ to mapping the transcriptional landscape of human embryonic stem cells^2^. Droplet-based scRNA-seq methods, such as Drop-seq^3^ and 10x Chromium^4^, are particularly powerful due to their high throughput: thousands of single cells can be analyzed in a single experiment. However, even with these high-throughput techniques, analyzing rare cell subpopulations remains a challenging task, often requiring protein-based enrichment for the subpopulation of interest before scRNA-seq^5,6^.

Many cell types require intracellular protein staining to be enriched. For example, Foxp3 is an intracellular marker of regulatory T cells^7^, and Oct4 and Nanog are intracellular reprogramming markers of induced pluripotent stem cells^8^. Intracellular protein staining requires cell fixation, which is most commonly achieved with paraformaldehyde (PFA) or methanol fixation. High-throughput techniques like Drop-seq and 10x Chromium have been shown to be compatible with methanol-fixed cells^9,10^. In many applications, however, PFA is preferred over methanol fixation due to the improved signal-to-background ratio in intracellular staining^11,12^, better preservation of intracellular structures’ integrity^13^, or simply because methanol fixation does not produce a signal^14^.

Important advances to scRNA-seq of PFA-fixed cells have recently been made. A well plate-based method^5^ was shown to be compatible with PFA-fixed cells, but the relatively low throughput nature of this method excludes its applicability from a wide range of problems that search for rare phenotypes in broad cellular populations. Most recently, a high-throughput scRNA-seq method that combines well plate-based combinatorial indexing and the 10x platform, called scifi-RNA-seq^15^, has been shown to work with formaldehyde-fixed single cells and single nuclei. However, scifi-RNA-seq requires a separate reverse transcription step before droplet encapsulation, thus complicating the sample processing step. Another method called inCITE-seq^16^ has been developed for sequencing formaldehyde-fixed single nuclei with 10x. In contrast to scifi-RNA-seq, inCITE-seq performs cross-link reversal and reverse transcription inside the droplets. Like scifi-RNA-seq, however, inCITE-seq requires laborious preprocessing of the samples. Because inCITE-seq has only been demonstrated on formaldehyde-fixed cell nuclei, most of the mature mRNA transcripts that reside in the cytoplasm cannot be measured. In summary, despite many technical advances, sequencing of PFA-fixed single cells continues be a complicated process with several shortcomings.

The ability to study PFA-fixed samples is important, particularly in virology. For example, Kaposi’s sarcoma-associated herpesvirus (KSHV), also known as human herpesvirus type 8 (HHV-8), is a human gammaherpesvirus that causes a number of malignancies such as Kaposi’s sarcoma, primary effusion lymphoma, and multicentric Castleman’s disease^17,18^. There is considerable interest in unraveling the molecular details of the host factors that modulate KSHV latency and reactivation, because both latency and low-level reactivation are known to contribute to viral tumorigenesis^19^, and therapeutic induction of reactivation could sensitize latently infected cells to currently available anti-herpesvirus drugs^20^. However, studying KSHV reactivation is challenging because of the low level of reactivation: only a small proportion of latently infected cells typically undergo reactivation, even when treated with known chemical inducing agents such as sodium butyrate (NaBut) and tetradecanoyl phorbol acetate (TPA)^17^. Single-cell transcriptomic analysis of reactivated cells, therefore, requires enrichment beforehand, so that the majority of sequencing reads are not spent on non-reactivated cells. Such enrichment would involve PFA fixation and intracellular staining of a viral protein marker.

Another use of PFA fixation is to inactivate infected cells or patient-derived materials. This would greatly facilitate the study of highly pathogenic virus-infected cells outside high-containment BSL-3 facilities, which are often not readily available. The importance of such flexibility has become evident during the ongoing COVID-19 pandemic.

Here we describe FD-seq (Fixed Droplet RNA sequencing), an easy-to-use, droplet-based high-throughput method for single-cell RNA sequencing of PFA-fixed, permeabilized, stained, and sorted whole cells. We show that FD-seq preserves the RNA integrity and relative transcripts abundances compared to Drop-seq for live cells, and that FD-seq yields a higher number of detected genes and transcripts than the methanol fixation method. By applying FD-seq to studying KSHV reactivation, we find that *TMEM119*, a gene with unknown functions, potentially plays a role in promoting KSHV reactivation. We then utilize FD-seq to study OC43 infection in fixed cells. OC43 is a human betacoronavirus that causes the common cold, and it has successfully been used to discover drugs that inhibit SARS-CoV-2 replication in vitro^21^. We find that after OC43 infection, a large subpopulation of cells express low levels of viral genes, but highly upregulate pro-inflammatory genes compared to cells with higher expression of viral genes. Our study shows that FD-seq is a valuable tool for studying rare cell subpopulations, and provides researchers with greater flexibility in choosing the cell fixation, permeabilization, or pathogen inactivation method.

## Results

### Development of FD-seq for sequencing of PFA-fixed single cells

To facilitate ease of adoption, we developed FD-seq based on Drop-seq^3^. In the standard Drop-seq protocol, single cells are partitioned with uniquely barcoded ceramic beads inside nanoliter droplets in oil, using a microfluidic device. The cells are individually lysed inside the droplets, and their mRNAs are captured by the oligonucleotides on the barcoded beads. Next, the droplets are broken to recover the beads, and the beads are extensively washed to remove uncaptured mRNAs. After pooling the beads, the captured mRNAs undergo reverse transcription, exonuclease I digestion to remove the free oligonucleotides on the beads, and whole transcriptome amplification. Finally, the barcoded and amplified complementary DNAs (cDNAs) are tagmented and sequenced.

PFA fixation of cells induces cross-linking between nucleic acids and proteins. To extract RNA from PFA-fixed cells, a cross-link reversal step through heating is therefore required. We reasoned that for FD-seq to be readily used without additional significant processing steps, cross-link reversal must be performed inside the droplets following droplet encapsulation.

**Fig. 1:**
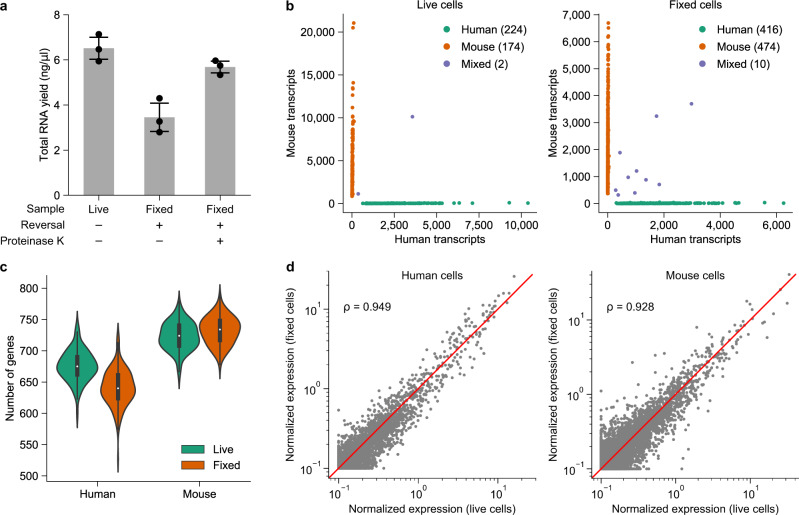
Benchmarking and validation of FD-seq. **a** Bar plots showing total RNA yield from bulk live cells, and bulk fixed cells that underwent cross-link heat reversal (1 h at 56 °C) with or without 40 U/mL of proteinase K. Data are presented as mean ± standard deviation. *n* = 3 technical replicates. **b** Species-mixing plots showing the single-cell capture efficiency of Drop-seq and FD-seq. The multiplet rate for live cells and fixed cells were ~0.5% and ~1%, respectively. Human BC3 cells were combined with mouse 3T3 cells at equal concentration, and processed with Drop-seq or FD-seq. See the “Methods” section for more details. **c** Violin plots and box plots showing the number of detected genes in live and fixed cells for each species. For this analysis, only cells with at least 1500 transcripts were considered, and 1000 transcripts were randomly sampled from each single cell. The white dots inside the violin plots represent the median of the data, the black boxes represent the first and third quartiles, and the black lines represent the values 1.5× the interquartile range beyond the first and third quartiles. *n* = 157 and 164 single cells for live and fixed human samples. *n* = 150 and 267 single cells for live and fixed mouse samples. **d** Comparison of the normalized expression level of each gene between live and fixed cells for each species (see “Methods” section). Each dot represents the average expression level of a gene, and the red line indicates the line *y* = *x*. The plots also show the Pearson’s correlation coefficient *ρ* of the log-normalized gene expression level between live and fixed cells for each species.

Therefore, we first tested different heating conditions for the cross-link reversal step at bulk level, followed by lysing the uncross-linked cells with Drop-seq lysis buffer, and total RNA extraction using a commercial kit (Fig. 1a, Supplementary Fig. 1, and see “Methods” section). We found that a 1-h incubation at 56 °C in the standard Drop-seq lysis buffer efficiently reversed PFA cross-linking, in agreement with the previous literature^5^. The RNA yield could be improved further by adding proteinase K to the lysis buffer at an optimal concentration of 40 U/mL (Fig. 1a and Supplementary Fig. 1a). Whereas proteinase K treatment did not significantly affect the RNA quality at any of the tested concentrations, consistently resulting in high-quality total RNA as demonstrated by the high RNA integrity numbers (above 8.0, Supplementary Fig. 1b, c), we observed that increasing the proteinase K concentration above 40 U/mL resulted in a reduction in the overall RNA yield and higher variability (Supplementary Fig. 1a).

Next, we compared the single-cell capture efficiency, and the extent of cross-droplet RNA contamination between the standard Drop-seq method on live cells, and the FD-seq method on PFA-fixed cells by performing species-mixing experiments (Fig. 1b). For Drop-seq, we analyzed a 1-to-1 mixture of live BC3 cells, a human primary effusion lymphoma (PEL) cell line, and mouse 3T3 cells. For FD-seq, we separately fixed BC3 and 3T3 cells with 4% PFA, permeabilized them with 0.1% Triton-X, and analyzed a 1-to-1 mixture of these two cell lines. The sequencing results were aligned to a combined human-mouse reference genome, and the number of human and mouse transcripts per single-cell barcode was then calculated. The rate of cell barcodes having both mouse and human transcripts, which indicates cross-droplet contamination and/or multiple cells captured in the same droplet, were very similar and minimally observed in both live and fixed cell samples (~0.5% and ~1% for live and fixed cells, respectively, Fig. 1b). This indicates that FD-seq has similar single-cell capture efficiency as Drop-seq.

We also observed that the number of genes detected in fixed cells was comparable to that of live cells across species (median of 640 genes in fixed cells compared to 675 genes in live cells for human cells, and median of 734 genes compared to 724 genes for mouse cells) (Fig. 1c). The relative expression levels of the detected genes were well correlated between live and fixed cells (Fig. 1d), even though fewer transcripts were detected in fixed cells than in live cells on average (Fig. 1b and Supplementary Fig. 2a). Finally, live and fixed cells showed a similar percentage of reads mapped to introns and exons (Supplementary Fig. 2b).

We also assessed the technical repeatability of FD-seq by analyzing two technical replicates of fixed and permeabilized A549 cells, a human lung epithelial cell line, with FD-seq. We found that the two replicates show strong agreement in terms of the number of detected transcripts and genes, and the relative gene expression level (Supplementary Fig. 3).

Taken together, these results demonstrate that FD-seq is a reliable method for the whole transcriptome analysis of PFA-fixed single cells. The performance of single-cell sequencing is maintained with fixed cells when FD-Seq is utilized, and FD-seq shows comparable performance to the standard Drop-seq protocol used for live, unfixed cells.

### PFA fixation detects a higher number of genes and transcripts compared to methanol fixation in single cells

We next investigated how PFA fixation in FD-seq compare to methanol (MeOH) fixation. Methanol fixation has recently been shown to be compatible with Drop-seq^9^ by introducing a suitable rehydration step before droplet generation. Here we fixed A549 cells with either PFA or methanol, and then analyzed the single-cell transcriptomes by RNA-seq using the FD-seq or Drop-seq protocol, respectively (see “Methods” section).

**Fig. 2:**
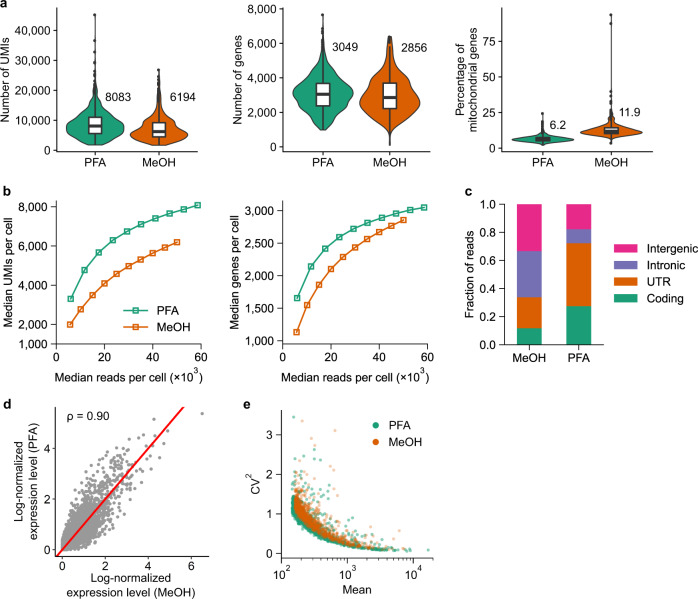
Comparison between PFA and methanol fixation shows higher gene and transcript recovery with FD-seq. **a** Violin and box plots of the number of UMIs, the number of genes, and the percentage of mitochondrial genes detected in single A549 cells for each fixation method. The PFA and methanol (MeOH) samples received ~58,600 and 50,000 median reads/cell, respectively. The numbers by the violin plot indicate the median values. The middle line inside the box indicates the median, the upper and lower edges of the box indicate the first and third quartiles, and the whiskers extend to 1.5× the interquartile range beyond the first and third quartiles. **b** The effects of sequencing depth on the number of detected UMIs and genes per cell. **c** The distribution of mapped reads to different genomic regions. **d** Correlation of the log-normalized average expression level of each gene between the two fixation methods (see “Methods” section). The plot also shows the Pearson’s correlation coefficient *ρ* between the two methods. The red line indicates *y* = *x*. **e** Technical variance of each gene estimated by the gene’s mean and squared coefficient of variation (CV^2^). In **a**–**e**, *n* = 999 and 498 single cells for PFA and methanol samples, respectively.

We found that PFA fixation resulted in a higher number of detected transcripts, or UMIs, (median 8083 compared to 6194 transcripts) and genes (median 3049 compared to 2856 genes), and a lower percentage of mitochondrial genes (median 6.2% compared to 11.9%) than methanol fixation (Fig. 2a). To determine the effect of sequencing depth on the number of UMIs and genes, we randomly sub-sampled the sequencing reads and found that PFA fixation consistently returned a higher number of UMIs and genes across different read depths (Fig. 2b). The effect of sequencing depth on the number of transcripts is much more pronounced: the difference between the two fixation techniques could reach almost 2000 UMIs. Compared to methanol fixation, FD-seq has a higher proportion of reads mapped to the coding and untranslated regions, and a lower proportion mapped to the intergenic and intronic regions (Fig. 2c). Despite this, the average gene expression levels were well correlated between the two fixation methods (Pearson’s correlation coefficient was ~0.90, Fig. 2d). Lastly, we estimated the technical variance of each method by calculating the gene-level coefficient of variation (CV)^22^. As expected, for both fixation methods, gene abundance negatively correlates with gene variance (Fig. 2e). In addition, both methods exhibited very similar technical variance. In summary, FD-seq was able to recover more genes and transcripts than methanol fixation, and both PFA-fixed cells and methanol-fixed cells showed similar average gene expression levels and technical variance.

### FD-seq reveals heterogeneity in KSHV reactivated single tumor cells

Having established the validity of FD-seq for single-cell transcriptome analysis of PFA-fixed cells, we applied FD-seq to test a hypothesis, that the heterogeneity in specific host genes contributed to the restricted KSHV reactivation in human primary effusion lymphoma (PEL) cells BC3, and to identify those genes.

**Fig. 3:**
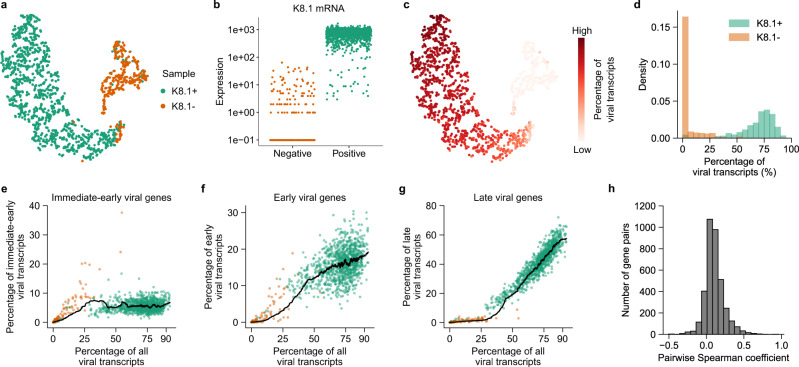
FD-seq reveals heterogeneity in KSHV viral reactivation. **a** t-SNE plot of K8.1+ (green, lytically reactivated) and K8.1− (red, non-reactivated) BC3 cells as analyzed by FD-seq. Clustering was performed using both host and viral transcripts. **b** KSHV K8.1 mRNA levels in the sorted K8.1− (non-reactivated) and K8.1+ (reactivated) subpopulations. **c** t-SNE plot of reactivated and non-reactivated cells as in **a**, colored by the percentage of total viral transcripts. **d** Histogram showing the percentages of detected transcripts that are from KSHV in K8.1+ and K8.1− BC3 cells. **e**–**g** Change in the percentages of **e** intermediate-early, **f** early, and **g** late viral genes as a function of the percentage of total viral transcripts. The black lines indicate the moving averages (50-cell window). **h** Histogram of pairwise Spearman correlation coefficients between viral genes.

To identify lytically reactivated BC3 cells, we used flow cytometry to measure the expression of the intracellular viral glycoprotein K8.1, which is only expressed during lytic reactivation. As expected, we did not observe any appreciable K8.1 expression in untreated BC3 cells (Supplementary Fig. 4a). Treatment with NaBut or TPA, which are known to induce KSHV reactivation^17^, resulted in a small fraction of cells expressing K8.1 (~8% compared to ~2.5%, respectively, Supplementary Fig. 4b). To enrich for reactivated cells, TPA-treated BC3 cells were fixed, permeabilized, stained for K8.1 viral protein, and sorted by fluorescence-activated cell sorting (FACS) based on the K8.1 expression level (2.2% of the cells were K8.1-positive, Supplementary Fig. 5), followed by single-cell transcriptome analysis with FD-seq. We obtained high-quality data for 1035 K8.1-positive (reactivated) single cells and 286 K8.1-negative (latent, non-reactivated) cells. High dimensional clustering and visualization showed a clear separation between reactivated and non-reactivated cells (Fig. 3a), and analysis of the *K8.1* mRNA level confirmed the enrichment of the K8.1+ cell subpopulation of interest (Fig. 3b). Moreover, the high proportion of viral transcripts in the K8.1+ subpopulation compared to the K8.1− population (69% and 4% on average, respectively) confirmed that the sorted population was indeed mostly composed of reactivated cells (Fig. 3c, d).

Herpesvirus reactivation involves the highly regulated, sequential expression of immediate-early, early, and late viral genes^23^ that was recapitulated in our FD-seq results (Fig. 3e–g). By ordering the cells by the percentage of viral genes relative to the total transcript content, we found that the relative abundance of immediate-early viral genes increased with total viral gene content, then plateaued after the total viral transcript abundance reached 50%. Early viral transcripts also increased early and monotonically with the abundance of total viral transcripts, without plateauing like the immediate-early genes. On the other hand, late viral transcripts were only detected in cells with more than 25% total viral transcripts, and increased strongly with higher total viral transcript abundance. Thus, FD-seq’s results are in agreement with the expected kinetics of viral gene expression obtained from population-averaged measurements, and suggest that the percentage of viral transcript content is a good indicator of the stage of KSHV reactivation.

Interestingly, we found that the K8.1+ population was highly heterogeneous in viral transcript expression: the proportion of viral transcripts among all detected transcripts varied from below 50% to over 90% (Fig. 3d), and the correlation between the expression levels of viral genes was very low, even between viral genes that have the same kinetics (Fig. 3h and Supplementary Fig. 6a). This agrees with two recent studies that showed heterogeneity in host cell factor abundance at the single-cell level in herpes simplex virus type 1 (HSV-1) infection^24^, and low correlation in expression of viral genes in a murine gammaherpesvirus infection model^25^. This may partially be caused by the possibility that the sorted K8.1+ cells were at different stages of reactivation. Indeed, flow cytometry analysis showed that K8.1 protein abundance within the positive population varied over one order of magnitude (Supplementary Fig. 5). However, this alone could not sufficiently explain the poor correlation between viral genes, because we also observed a low correlation between cells in the same stages of reactivation (i.e., cells with similar viral transcript abundance) (Supplementary Fig. 6b).

In short, we successfully applied FD-seq to characterize KSHV-infected cells undergoing reactivation from latency, revealing the highly heterogeneous nature of this process.

### FD-seq shows that TMEM119 facilitates KSHV reactivation

Next, we sought to identify host genes that facilitate KSHV reactivation by looking for differentially expressed host genes that are positively correlated with viral transcript abundance. We only looked for positively correlated host genes in our analysis, because the majority of differentially expressed genes were negatively correlated with the relative abundance of viral transcripts (Supplementary Fig. 7a). While this could suggest that their downregulation promotes KSHV reactivation in the cells^26^, the lower expression level measured in these genes could be due to undersampling caused by the possibility that sequencing reads are mostly used by the highly abundant viral transcripts in the K8.1+ subpopulation (which can account for up to 96% of all detected transcripts, Fig. 3d).

**Fig. 4:**
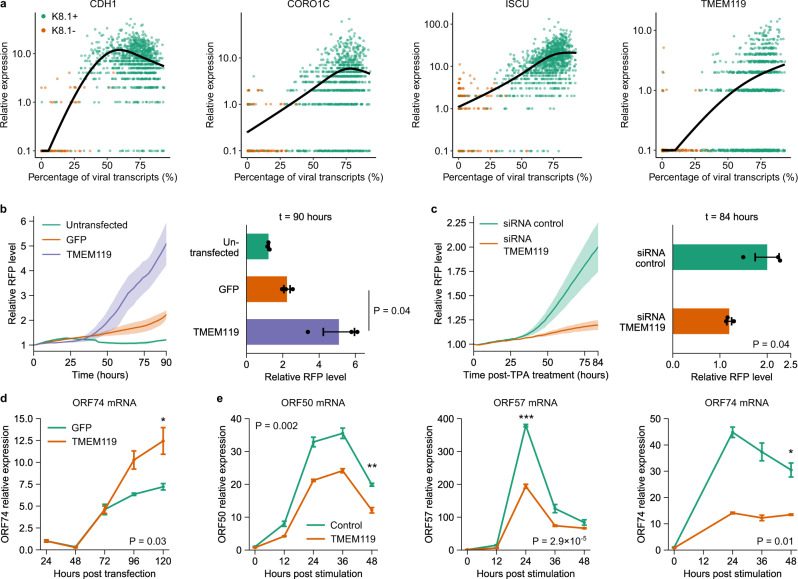
FD-seq identifies *TMEM119* as a potential host factor that mediates KSHV reactivation. **a** Plots showing the relative expression of the four host factors *CDH1*, *CORO1C*, *ISCU*, and *TMEM119* (based on the genes’ normalized counts) as a function of the percentage of KSHV transcripts. Each dot indicates a single cell. **b** Live-cell imaging analysis of RFP expression, which is indicative of KSHV reactivation level, in untransfected HEK293T.rKSHV219 cells or cells transfected with *TMEM119* or GFP control (left), and end-point quantification of RFP expression at 90 h (right). **c** Live-cell imaging analysis of RFP expression in HEK293T.rKSHV219 cells transfected with control or *TMEM119*-targeting siRNAs for 48 h, followed by treatment with 2 ng/mL TPA (left), and end-point quantification of RFP expression at 84 h (right). In **b**, **c**, the lines indicate the mean, and the ribbons indicate the s.e.m. of the data. **d** Time-course RT-qPCR analysis of KSHV *ORF74* abundance in HEK293T.rKSHV219 cells transfected with *TMEM119* or GFP control. **e** Time-course RT-qPCR analysis of KSHV *ORF50*, *ORF57*, and *ORF74* levels in HEK293T.rKSHV219 cells transfected with control or *TMEM119*-targeting siRNAs. **b**–**e** Data are represented as mean ± s.e.m. *n* = 3 biological replicates per sample. **d**, **e** *P < 0.05, **P < 0.01, ***P < 0.001 (one-sided Welch’s *t*-test).

We found four host genes whose expression positively correlated with the level of KSHV transcripts: *ISCU*, *CDH1*, *CORO1C*, and *TMEM119* (*q*-value < 10^−100^ for all four genes, Fig. 4a and Supplementary Data 1). We observed that the expression of *ISCU* was relatively low in non-reactivated cells, and increased significantly with the abundance of viral transcripts. On the other hand, *CDH1*, *CORO1C* and *TMEM119* were mostly undetectable in non-reactivated cells, and increased by 1–2 orders of magnitude in cells expressing high levels of viral transcripts. We also confirmed the upregulation of *ISCU*, *CORO1C,* and *TMEM119* following KSHV reactivation in bulk cell samples by qPCR (Supplementary Fig. 7b, c).

To determine whether the strong correlation between these host transcripts and viral gene expression means that these genes modulate KSHV reactivation induction and/or efficiency, we next tested the effect of their overexpression on KSHV reactivation. To this end, we used live-cell imaging to monitor KSHV reactivation in HEK293T.rKSHV219 cells following exogenous expression of *CDH1*, *CORO1C*, *ISCU*, or *TMEM119*. HEK293T.rKSHV219 are HEK293T cells that have been latently infected with the recombinant KSHV.219 virus strain, which constitutively expresses GFP, and further encodes RFP under control of the viral lytic PAN promoter^27^. Overexpression of *CDH1*, *CORO1C*, or *ISCU* had no significant effect on KSHV reactivation efficiency. On the other hand, we observed a clear increase in RFP expression, which is indicative of viral reactivation, upon exogenous expression of *TMEM119* relative to a GFP-encoding control vector (Fig. 4b and Supplementary Fig. 8a–c). Conversely, silencing of endogenous *TMEM119* significantly reduced the level of TPA-induced KSHV reactivation (Fig. 4c and Supplementary Fig. 8b, c). These results were corroborated by RT-qPCR analysis of KSHV lytic gene expression, which showed that *TMEM119* overexpression enhanced KSHV *ORF74* expression compared to the *GFP* control (Fig. 4d), while *TMEM119* silencing led to a reduction in KSHV *ORF50, ORF57, and ORF74* expression (Fig. 4e). Together, these results showed that *TMEM119* positively modulates KSHV reactivation efficiency.

### FD-seq reveals pro-inflammatory signatures in a subpopulation of OC43-infected cells

We next applied FD-seq to study the infection of the betacoronavirus OC43 in single human lung cells. OC43 is a human pathogen that causes the common cold, and is a close relative of SARS-CoV-2. We have recently shown that many drugs that inhibit the replication of OC43 also inhibit SARS-CoV-2 replication in vitro^21^, suggesting that OC43 is a good model system for SARS-CoV-2 infection.

**Fig. 5:**
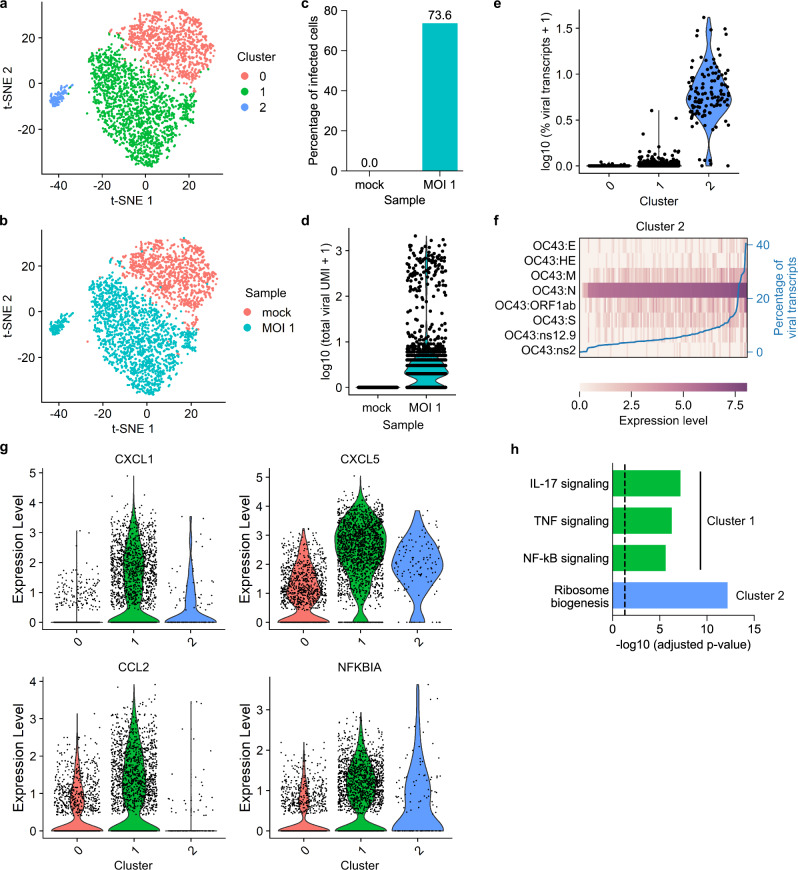
Single-cell heterogeneity and pro-inflammatory signatures after coronavirus OC43 infection. **a**, **b** t-SNE plots with cells colored by **a** cluster identity or **b** sample type. **c** Bar plot showing the percentage of infected cells (defined as cells that expressed at least 1 detected viral transcript) of mock-infected and MOI 1 sample. **d** Violin plot showing the distribution of total viral transcript counts. **e** Violin plot showing the distribution of the percentage of the total viral transcript by cluster identity. **f** Heatmap showing the relative expression level of each viral gene in each single cell in cluster 2. The blue line shows each cell’s percentage of total viral transcripts. **g** Violin plots showing the expression of four representative immune-related genes that are upregulated in cluster 1. The expression levels in **f**, **g** were log-normalized transcript counts. **h** Bar plot showing the multiple comparison corrected *P*-values (Fisher’s one-tailed test with g:SCS correction^32^) of upregulated KEGG pathways in clusters 1 and 2. The vertical dashed line indicates *P*-value = 0.05.

First, we infected A549 cells with OC43 at a multiplicity of infection (MOI) of 1, fixed the cells with PFA, and performed FD-seq on mock-infected and OC43-infected cells. We obtained 1167 and 1924 high-quality single cells from mock-infected and OC43-infected cells, respectively. After regressing out the effects of cell cycle variations (Supplementary Fig. 9a, b), high dimensional clustering yielded three main clusters of cells (Fig. 5a). Cluster 0 mainly contained the mock-infected cell, while clusters 1 and 2 mainly contained OC43-infected cells (Fig. 5b). At an MOI of 1, more than 70% of the cells showed some level of viral gene expression (Fig. 5c). Interestingly, this infected population consisted of two subpopulations, clearly separated by the number of detected viral transcripts: a larger subpopulation expressing a low number of viral transcripts (below 100 viral transcripts, median 2 transcripts) and a smaller subpopulation expressing a much higher number of viral transcripts (above 100 viral transcripts, median 428 transcripts) (Fig. 5d). This small subpopulation of highly infected cells corresponded to cluster 2 (Fig. 5e and Supplementary Fig. 9c, d).

Next, we investigated the expression profile of OC43 viral genes as a function of total viral gene level within cluster 2’s cells. Gene *N* (which encodes the viral nucleocapsid protein^28^) was uniformly highly expressed (Fig. 5f). The expression of the remaining viral genes was more heterogeneous, with *ORF1ab*, *M* (membrane gene), and *S* (spike gene) being more highly expressed overall. These patterns of viral gene expression level were also observed in all infected cells (Supplementary Fig. 10).

On the other hand, cluster 1 consisted of cells that were exposed to the virus but failed to express high levels of viral genes (Fig. 5d and Supplementary Fig. 9c, d). Interestingly, this cluster is also enriched in pro-inflammatory genes, such as *CXCL1*, *CXCL5*, *CCL2,* and *NFKBIA* (Fig. 5g, Supplementary Fig. 11, and Supplementary Data 2). *CXCL1* and *CXCL5* encode the protein ligands of the CXC chemokine receptor 2 (CXCR2), and are crucial to neutrophil recruitment^29^. *CCL2* encodes a different chemokine that is responsible for monocyte recruitment from the bone marrow^30^. *NFKBIA* encodes the inhibitor IκBα to the transcription factor NF-κB, which is an important regulator of the immune response^31^. KEGG pathway analysis^32^ showed that cluster 1 was enriched for three main pro-inflammatory pathways: TNF, IL-17, and NF-κB signaling pathways (Fig. 5h). Cluster 2 was enriched for the ribosome biogenesis pathway, suggesting an increased need for protein biogenesis during OC43 replication.

In short, FD-seq revealed that the majority of cells did not express a high level of viral genes after exposure to OC43 at MOI of 1, and that these cells upregulated pro-inflammatory genes. These findings are in agreement with our previous characterization of HSV-1 infection at the single-cell level^24^, which showed that most HSV-1 infected cells did not express high levels of viral genes.

## Discussion

Here we present FD-seq, a method for high-throughput droplet-based RNA sequencing of PFA-fixed, permeabilized, stained, and sorted single cells. FD-seq is particularly useful for sequencing rare cell subpopulations that require intracellular staining and FACS-enrichment, and for rendering infectious samples safe for handling. Although Drop-seq has been shown to work with methanol fixation^9^, we demonstrated that FD-seq performs better than methanol fixation by detecting more genes and more transcripts. FD-seq will increase the flexibility for researchers in using high-throughput scRNA-seq, because PFA fixation has been shown to be better than methanol fixation for many applications^11–14^, and because PFA is a very commonly used fixative.

One problem with RNA sequencing of PFA-fixed cells is the need for cross-link reversal, which is usually done through heating. To avoid cross-cell contamination, the reversal step has to be done while cells are still inside the droplets. scifi-RNA-seq^15^ circumvented this challenge of cross-link reversal by performing reverse transcription with permeabilized cells and nuclei on a 96-well or 384-well plate before droplet generation, at the cost of higher experiment complexity. inCITE-seq^16^ also took an approach similar to FD-seq’s, by performing both cross-link reversal and reverse transcription inside the droplets after 10x droplet encapsulation. However, inCITE-seq was specifically optimized for single nuclei samples, and therefore cytoplasmic antibody staining signal and mature mRNAs are not detectable. In addition, inCITE-seq cannot use proteinase K like FD-seq, because with the 10x platform, reverse transcription is performed inside the droplets, and therefore the reverse transcriptase would have been digested had the proteinase K been added to the lysis buffer.

We applied FD-seq to study the process of KSHV lytic reactivation at the single-cell level. We found that reactivation is a very heterogeneous process, and that the expression levels of viral genes correlated poorly with one another. Additionally, we found the expression level of four host factors, namely *CDH1*, *CORO1C*, *ISCU*, and *TMEM119*, to be significantly positively correlated with viral reactivation. Using live-cell imaging and time-course study, we found *TMEM119* to have the strongest effect among these four genes in enhancing the degree of viral reactivation. Further studies are required to determine the mechanistic details behind the effects of *TMEM119* on KSHV reactivation.

Furthermore, we used FD-seq to study the host response of human lung cells to OC43 infection, which is a betacoronavirus, and the subsequent expression of viral genes at the single-cell level. First, we infected the cells with the virus, then fixed them with PFA, thereby inactivating the virus for safer sample handling, before analyzing the cells with FD-seq. We showed that most OC43-infected cells were unable to support a high level of viral gene expression, and instead upregulated pro-inflammatory signaling pathways. Because the cells were analyzed 4 days post-infection, it is not possible to determine whether these cells, which expressed a low level of viral genes, were abortively infected cells, or if they were infected through secondary infection. The investigation of the cellular mechanisms that lead to this heterogeneous infection outcome, and the biological functions of the upregulated pro-inflammatory genes, require further studies.

Taken together, these applications of FD-seq show that it is a valuable tool in the field of virology, and any other biological applications that require PFA fixation. Moreover, FD-seq can also be used for integrating protein activity with transcriptome information. For example, transcription factor expression levels and phosphorylation can be integrated with whole transcriptome analysis by combining intracellular protein staining with FD-seq. Furthermore, FD-seq is compatible with RNA velocity analysis, which relies on the detection of unspliced mRNA molecules^33^ (Supplementary Fig. 12). Finally, we anticipate that FD-seq could serve as a basis for the development of methods that allow sequencing of formalin-fixed paraffin-embedded (FFPE) tissues, due to the similarity between PFA fixation and FFPE, thereby enabling high-throughput single-cell sequencing of readily available samples in tissue banks.

## Methods

### Microfluidic device fabrication

The microfluidic device was fabricated based on the AutoCAD file provided in Macosko et al.^3^. The mold was made from SU8-3050 photoresist of ~110 µm in height. Then, polydimethylsiloxane (PDMS, Momentive RTV615) was cast on the mold to form the microfluidic devices. Plasma bonding was used to bond the PDMS device to a glass slide, and the microfluidic channels were coated with Aquapel.

### Cell culture

HEK293T.rKSHV219 cells were maintained in Dulbecco’s Modified Eagle’s Medium (DMEM) supplemented with 10% (v/v) fetal bovine serum (FBS), 2 mM GlutaMAX (Gibco), 1% (v/v) penicillin–streptomycin (P/S, Gibco), and 1 µg/mL puromycin. HEK293T.rKSHV219 cells were generated by infecting HEK293T cells with rKSHV.219^27^, and selecting with 1 µg/mL puromycin. The KSHV-positive human PEL cell line BC3 (ATCC) was cultured in Roswell Park Memorial Institute (RPMI) supplemented with 20% (v/v) FBS, 2 mM GlutaMAX, and 1% (v/v) P/S. A549 cells (ATCC) or A549 cells with H2B-Ruby fusion^21^ were maintained in DMEM supplemented with 10% FBS, without antibiotics. The 3T3 cells (p65^−/−^ 3T3 mouse embryonic fibroblast cells expressing p65-DsRed and H2B-GFP nucleus marker^31^) were cultured in DMEM supplemented with 10% (v/v) fetal bovine calf serum (HyClone), 1% (v/v) GlutaMAX, and 1× P/S.

### Optimization of RNA extraction condition for fixed and permeabilized cells

BC3 cells were harvested, centrifuged at 300–400 × *g* for 3 min to remove the cell media, and washed once with 1 mL of 1% BSA (molecular biology grade, Gemini Bio-Products) in PBS. Cells were fixed by adding 1 mL of 4% PFA in PBS (Santa Cruz Biotechnology) and incubated at room temperature for 15 min. Next, cells were centrifuged at 400 × *g* for 3 min, the paraformaldehyde was discarded, and cells were washed once with 1 mL of wash buffer (PBS with 1% BSA and 40 U/mL of RNase inhibitor, murine (NEB)). Cells were permeabilized by adding 500 µL of 0.1% Triton X-100 (molecular biology grade, Acros Organics) diluted in the wash buffer, and incubated at room temperature for 15 min. Then, 1 mL of wash buffer was added directly to the cells, cells were pelleted, the supernatant was discarded, and the cells were washed once more with 1 mL of wash buffer. To mimic the condition of antibody staining during optimization experiments, cells were resuspended in wash buffer and incubated on ice for 1 h, washed twice with the wash buffer, and finally kept on ice in the wash buffer before RNA extraction.

To serve as a positive control, RNA from bulk live cells was extracted using the RNeasy Plus Mini Kit (Qiagen). Cells were first suspended in a combination of the standard Drop-seq lysis buffer with PBS at 1-to-1 volume ratio (to make the lysis buffer’s final concentration the same as that in the droplets), incubated at room temperature for 10 min, and placed on ice for at least 5 min. Next, the cell lysate was combined with 350 µL of RLT plus buffer from the RNeasy kit, and processed according to the manufacturer’s protocol.

To extract RNA from bulk fixed cells, fixed and permeabilized cells (as described above) were suspended in a 1-to-1 mixture of PBS and Drop-seq lysis buffer, the latter of which was spiked in with various concentrations of proteinase K (NEB). Next, the sample was at 56 °C for 1 h, left at room temperature for 10 min, and placed on ice for at least 5 min. Finally, the cell lysate was combined with 350 µL of RLT plus buffer, and processed according to the RNeasy Plus Mini Kit’s protocol. The extracted RNA is then quantified using BioAnalyzer.

### Treatment of BC3 cells, flow cytometry, and FACS-sorting of reactivated cells

BC3 cells were mock-treated or treated with a final concentration of 20 ng/mL tetradecanoyl phorbol acetate (TPA, Millipore Sigma) for 36 h to induce KSHV reactivation. Cells were pelleted by centrifugation at 300 × *g* for 3 min and fixed with 4% PFA in PBS for 15 min at room temperature, followed by permeabilization with 0.1% (v/v) Triton X-100 in PBS with 1% (w/v) BSA for 15 min at room temperature. After washing and blocking the cells in PBS supplemented with 4% BSA, staining was performed with an anti-KSHV K8.1 antibody (sc-65446, Santa Cruz, 1:50 dilution) and an Alexa Fluor 488-conjugated goat-anti-mouse secondary antibody (A10667, Life Technologies, 1:4000 dilution) in PBS with 1% BSA and 40 U/mL RNase inhibitor, murine (NEB). K8.1+ and K8.1− cell subpopulations were analyzed on an LSRFortessa flow cytometer (BD Biosciences) and/or sorted on a FACSAriaIIIu cell sorter (BD Biosciences). FACS Diva and FlowJo software was used to analyze the flow cytometry data.

### Infection of A549 cells

OC43 (ATCC) were grown and titrated on A549 cells. For FD-seq experiments, A549 cells were seeded in 6-well plates and infected with OC43 at an MOI of 1 the following day. Cells were incubated at 33 °C for 4 days and were subsequently harvested for FD-seq.

The MOI was calculated as follows. A549 cells were infected with 10-fold serial dilutions of OC43. The virus was allowed to adsorb for 1 h at 33 °C, after which the inoculum was removed and the cells were overlaid with DMEM, 2% FCS, and 2% Cellulose. Infected cells were incubated at 33 °C for 2 weeks and subsequently fixed and stained with a 1:1 dilution of methanol:crystal violet. Plaques were counted and the virus stock concentration was determined in units of PFU/mL. The MOI reflects the ratio of PFU to the number of infected cells.

### FD-seq protocol

A step-by-step protocol for FD-seq is available on Protocol Exchange^34^. The sequences of the primers used in Drop-seq and FD-seq are listed in Supplementary Table 1.

The protocol for FD-seq is based on the McCaroll lab’s online Drop-seq protocol^3^. In summary, there are only two significant differences: 40 U/mL proteinase K was added to the Drop-seq lysis, and a heating step was added between the droplet generation and droplet breakage steps. First, the barcoded beads (ChemGenes Corporation, catalog number Macosko-2011-10(V+)) were suspended at 120,000 beads/mL in a modified Drop-seq lysis buffer: 200 mM Tris pH 7.5, 6% Ficoll type 400, 0.2% Sarkosyl, 20 mM EDTA, 50 mM DTT and 40 U/mL proteinase K (NEB). Cells were suspended in PBS with 0.01% BSA at 100,000 cells/mL. For droplet generation, the cells, beads, and oil (Bio-Rad, catalog number 1864005) were injected at flow rates of 3, 3, and 12 mL/h, respectively, using syringe pumps. After droplet generation, the droplets were heated on a heat block at 56 °C for 1 h to reverse the PFA cross-links, then incubated at room temperature for 10 min and kept on ice for at least 5 min. After this, the droplets were broken, the beads were collected, washed, and subjected to reverse transcription (using TSO_RNAhybrid primer) and exonuclease I digestion as per standard Drop-seq protocol.

For whole transcriptome amplification (WTA), the beads were distributed into a 96-well plate such that each well contained 5000 beads. A 50 µL PCR reaction was set up in each well using 1× KAPA HiFi Hotstart Readymix, and 0.8 µM TSO_PCR primer. The following thermal cycle program was used: 95 °C 3 min; 4 cycles of 98 °C 20 s, 65 °C 45 s, 72 °C 3 min; 10 cycles of 98 °C 20 s, 67 °C 20 s, 72 °C 3 min; 72 °C 5 min; 4 °C hold. After PCR, the WTA products were purified with 0.6× Ampure XP beads (Beckman Coulter), pooled and quantified with TapeStation (Agilent) or Qubit (Invitrogen).

To prepare the sequencing library, 450 pg of WTA products were incubated with the tagment buffer and tagment enzyme (Nextera XT DNA Library Prep Kit, Illumina) at 55 °C for 5 min. Next, the neutralization buffer was added, and the sample was incubated at room temperature for 5 min. After this, the Nextera PCR Master Mix and PCR primers P5-TSO_Hybrid and Nextera_N70X were added, and the sample was thermocycled as following: 95 °C 30 s; 12 cycles of 95 °C 10 s, 55 °C 30 s, 72 °C 30 s; 72 °C 5 min; 4 °C hold. Finally, the tagmentation products were purified with 0.6× Ampure XP beads, and quantified with TapeStation, BioAnalyzer, or Fragment Analyzer.

### Drop-seq protocol

The standard Drop-seq protocol is the same as the protocol for FD-seq as described above. The only two differences are that the lysis buffer does not include proteinase K, and the droplets are not subjected to the heating step between droplet generation and droplet breaking.

### Species-mixing experiment

Species-mixing experiments of fresh live samples were performed using human BC3 and mouse 3T3 cells, combined at 50,000 cells/mL/cell type. The samples were then processed with the standard Drop-seq protocol. For the species-mixing experiment of fixed samples, human BC3 and mouse 3T3 cells were separately fixed with PFA and permeabilized as described above. Then, the two cell types were combined at 50,000 cells/mL/cell type, and processed with the FD-seq protocol.

### Technical replication experiment

Two identical samples of A549-H2B-Ruby cells were separately fixed with PFA and permeabilized and permeabilized as above, and then processed with FD-seq on the same day.

### Methanol fixation experiment

A549 cells were harvested and split into two samples of ~1 million cells each, one for PFA fixation and FD-seq, and one for methanol fixation and Drop-seq. PFA fixation was performed as above. For methanol fixation^9^, cells were washed once with 1 mL of 1% BSA, then resuspended in 200 µL of ice-cold PBS. Next, ice-cold 100% methanol was added dropwise into the cells while gently vortexing the cells, and the cells were incubated on ice for 15 min. To rehydrate the cells, they were centrifuged at 1000 × *g* for 5 min, methanol was discarded, and cells were washed twice with 1 mL of 0.01% BSA in PBS. The rehydrated cells were then counted, resuspended at 100,000 cells/mL in 0.01% BSA in PBS, and processed according to the standard Drop-seq protocol.

### Plasmids and transfections

Expression plasmids encoding human *CDH1*, *TMEM119*, *ISCU*, and *CORO1C*, as well as KSHV *ORF50* and nls-eGFP under control of the human EF1α promoter in the pLV backbone were ordered from VectorBuilder. All sequences were verified by Sanger sequencing. HEK293T.rKSHV219 cells were reverse transfected with 0.8 μg plasmid DNA using Lipofectamine2000 (Life Technologies) following the manufacturer’s instructions, and seeded in 12- or 24-well plates for analysis by live-cell imaging or RT-qPCR as indicated. For silencing experiments, HEK293T.rKSHV219 cells were reverse transfected with 40 nM siRNA targeting *TMEM119* (Dharmacon siGenome SMARTpool M-018636-01-0005), *CDH1* (M-003877-02-0005), *CORO1C* (M-017331-00-0005), ISCU (M-012837-03-0005), or non-targeting control (D-001206-14-05) in 12- or 24-well plates using Lipofectamine RNAiMAX reagent (Life Technologies) following the manufacturer’s protocol. After 48 h, the cells were treated with 2 ng/mL TPA as indicated, and KSHV reactivation efficiency was assessed by live-cell imaging or RT-qPCR.

### Reverse transcription and real-time PCR (RT-qPCR)

Total RNA was extracted by using the HP Total RNA Kit (OMEGA Bio-Tek) using the manufacturer’s instructions. RT-qPCR was performed using either a one-step or a two-step protocol. For the one-step protocol, RT-qPCR was performed with equal amounts (25–500 ng) of RNA using the SuperScript III Platinum One-Step qRT-PCR kit with ROX (Thermo Fisher Scientific) on a 7500 Fast Real-Time PCR Machine (Applied Biosystems). Premixed master mixes containing TaqMan primers and probes for each individual gene were purchased from IDT (*GAPDH*, *TMEM119*, *CORO1C*, *ISCU*, and *CDH1*) or Applied Biosystems (18S). For the two-step protocol, reverse transcription was performed using the ProtoScript II First Strand cDNA Synthesis Kit (NEB) according to the manufacturer’s protocol. The kit’s oligo-dT primer was used as the primer in this step. Next, the RT products were diluted by 20-fold in water, and used as the input for qPCR (Luna Universal qPCR Master Mix, NEB). The primers’ sequences are given in Supplementary Table 2. The relative expression level of each target gene was calculated by normalizing for GAPDH or 18S levels using the Comparative Ct Method (ΔΔCt Method), and presented relative to the control sample.

### Live-cell imaging experiments

HEK293T.rKSHV219 cells were seeded in 24-well plates and transfected with plasmids for overexpression or siRNA for knock-down experiments. Cells were then imaged on a Nikon Ti-Eclipse containing an environmental chamber (37 °C, 100% humidity, 5% CO_2_). Images were acquired every 4 h for 4 days.

### Sequencing and alignment

Drop-seq and FD-seq libraries were sequenced on a NextSeq 550 machine with the following read distribution: 20 bp for read 1, 60 bp for read 2, and 8 bp for index 1 read. The custom read 1 sequencing primer Read1CustomSeqB was used in place of Illumina’s read 1 primer (Supplementary Table 1). Alignment was performed using Picard version 2.21.8 (https://github.com/broadinstitute/picard), Drop-seq tools version 1.13 or version 2.3, and STAR aligner version 2.6.1b^35^. The valid cell barcodes were chosen using the knee plot method. The species-mixing sequencing results were aligned to the combined human-mouse reference genomes (accession number GSE63269). Alignment was performed to a concatenated version of the human genome with KSHV genome GQ994935.1^36^, or the human genome GRCh38 and OC43 genome NC_006213.1^28^.

### Read subsampling and read mapping

In the FD-seq and methanol fixation comparison experiment, we used samtools view command^37^ to subsample the output BAM file from Drop-seq tools version 2.3 DetectBeadSynthesisErrors command. To calculate the proportion of reads mapped to different genomic regions, we used Picard CollectRnaSeqMetrics command on the output BAM file directly from the STAR aligner. The refFlat file for the GRCh38 reference genome was downloaded from http://hgdownload.cse.ucsc.edu/goldenPath/hg38/database/.

### scRNA-seq data analysis

Data analysis was done using Python 3, RStudio, and R packages Monocle 2^38–40^q and Seurat 3^41,42^. Data visualization was done using R and Python.

For species-mixing data, a cell is classified as human or mouse if more than 90% of the detected transcripts are mapped to human or mouse, respectively. Otherwise, the cell is considered mixed. dropEst pipeline^43^ (version 0.8.5) was used with argument –L eiEIBA to annotate reads mapping to exon, intron, or exon/intron spanning regions. To compare the mean expression level between live and fixed samples of the same species, we normalized the single-cell UMI counts using Monocle’s estimateSizeFactors function, then calculated the average of each gene in each sample. Any average value below 0.1 is set to 0.1 for plotting.

For the technical replication experiment, we first removed cells with fewer than 1000 UMIs or more than 15,000 UMIs, and discarded genes detected in fewer than 5 cells. We then imported the count data into Seurat 3, normalized, and scaled the data with the default settings. To compare the gene expression level between the two technical replicates, we calculated the average normalized expression using Seurat’s AverageExpression function, then plot the natural logarithm of the average expression with one added pseudocount.

For the FD-seq and methanol fixation comparison experiment, we first discarded cells with lower than 1000 UMIs and genes detected in fewer than 5 cells. We then used Seurat 3 to log-normalize and scale the data. To compare the two fixation methods, we performed similar calculations as for the technical replication experiment above. To calculate the technical variance^22^, we normalized the UMI count by converting it to UMIs per million, then randomly chose 400 cells from each fixation method and used the 1000 most highly expressed non-mitochondrial genes to calculate the mean and squared coefficient of variation.

For the KSHV reactivation experiment, we first discarded cells with more than 3000 genes detected, and discarded cells with fewer than 1000 UMIs or more than 10,000 UMIs. Then we discarded genes detected in fewer than 5 cells. To find the relative expression of each gene, we normalized the transcript counts using estimateSizeFactors and estimateDispersions functions in Monocle 2. To cluster the cells, we first performed dimension reduction with t-SNE using the first 4 principal components, then clustered the cells by setting rho_threshold = 17 and delta_threshold = 11. We then set the percentage of viral transcripts as the pseudotime parameter, and performed a pseudotime analysis in order to find genes correlated with the abundance of viral transcripts.

For the OC43 infection experiment, we discarded cells with fewer than 1000 UMIs or more than 20,000 UMIs, and discarded genes detected in fewer than 5 cells. We then used Seurat 3 to remove the cell cycle effects (using CellCycleScoring function), log-normalize and scale the data. The log-normalized counts are used to show gene expression level. To cluster the cells, we used the first 10 principal components for the FindNeighbors and RunTSNE functions, and resolution = 0.2 for the FindClusters function. To find the cluster markers, we used min.pct = 0.25 and logfc.threshold = 0.25 settings for the FindAllMarkers function. To find enriched KEGG pathways in each cluster, we used g:Profiler^32^ (https://biit.cs.ut.ee/gprofiler/gost) with the default settings on the list of genes upregulated in each cluster as found by Seurat’s FindAllMarkers function.

### RNA velocity analysis

The output files of Drop-seq tools DetectBeadSynthesisErrors function were processed with the dropEst pipeline^43^ (version 0.8.5) to tag spliced and unspliced transcripts, and the results were analyzed with the Python’s velocyto package^33^ (version 0.17.17).

### Reporting summary

Further information on research design is available in the Nature Research Reporting Summary linked to this article.

## Supplementary information

Supplementary information

Description of Additional Supplementary Files

supplementary data 1

supplementary data 2

Reporting Summary

## Data Availability

The raw sequencing data and UMI count data generated in this study have been deposited in NBCI’s Gene Expression Omnibus under accession number GSE156988. The human-mouse combed reference genome is available under accession number GSE63269. GQ994935.1 and NC_006213.1 were used as the reference genomes for KSHV and OC43 viral genes, respectively. Source data are provided with this paper.

## Source data

Source Data

## Code availability

The code used for sequencing alignment and data analysis is available on Github: https://github.com/tay-lab/FD-seq^44^.
