# Supplementary material for: High-throughput RNA sequencing of paraformaldehyde-fixed single cells
Source: Nat Commun. 2021 Sep 24;12:5636. doi: 10.1038/s41467-021-25871-2 (PMC8463713; doi:10.1038/s41467-021-25871-2)
Supplement: Supplementary file 1 — Supplementary information [file 41467_2021_25871_MOESM1_ESM.pdf]

Supplementary Information

High-throughput RNA sequencing of paraformaldehyde-fixed single cells

Hoang Van Phan,[1*] Michiel van Gent,[2,3*] Nir Drayman,[1*] Anindita Basu,[4] Michaela U. Gack,[2,3] Savaş Tay[1]

[1]Pritzker School of Molecular Engineering, The University of Chicago, Chicago, IL, USA

[2]Department of Microbiology, The University of Chicago, Chicago, IL, USA

[3]Florida Research and Innovation Center, Cleveland Clinic, Port Saint Lucie, FL, USA

[4]Department of Medicine, The University of Chicago, Chicago, IL, USA

*These authors contributed equally

Corresponding author: tays@uchicago.edu

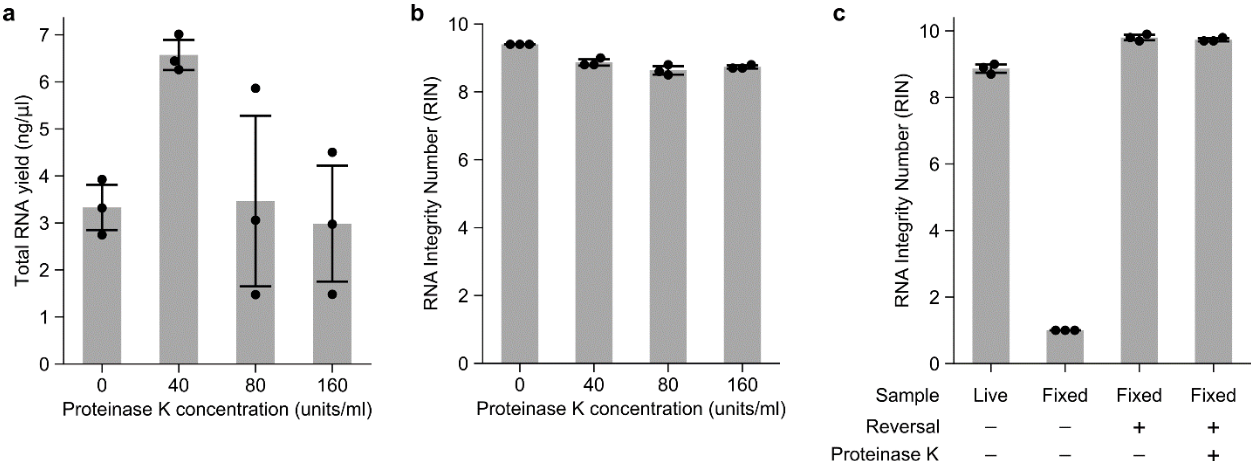

Supplementary Figure 1. Optimization of total RNA extraction from bulk fixed cells. (a, b) Total RNA yield (a) and RNA integrity number (RIN) (b) at different proteinase K concentrations. **(c)** RINs of RNA extracted from fresh live cells, of RNA extracted from fixed cells without heat reversal and without using proteinase K, and of RNA extracted from fixed cells after heat reversal, and with and without using proteinase K. Data is presented as mean ± standard deviation. n=3 technical replicates for all samples.

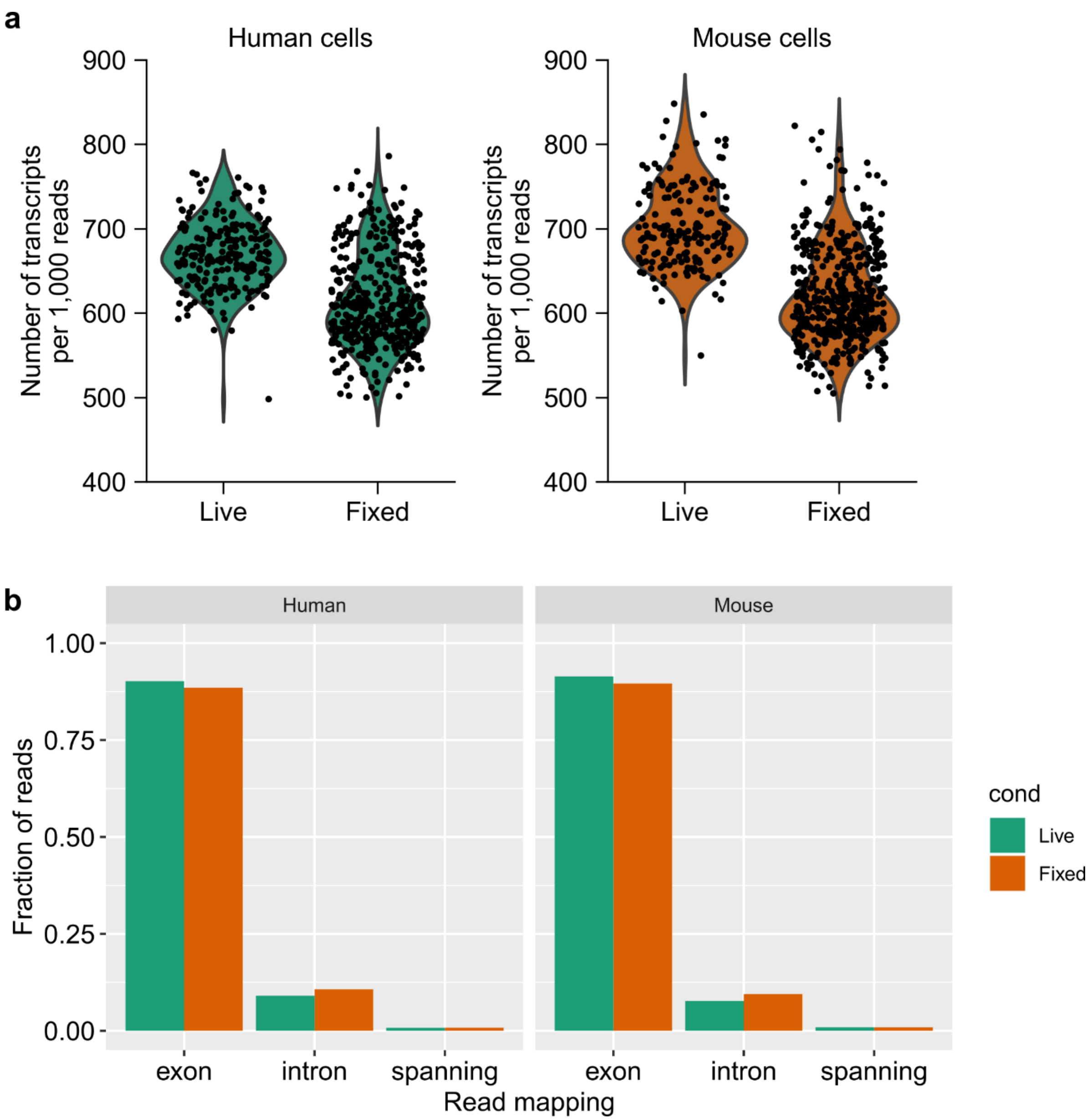

Supplementary Figure 2. Comparison between number of transcripts discovered and exon/intron mapped reads in fresh live and fixed cells. (a) The number of transcripts per 1,000 reads detected. **(b)** The fraction of reads mapped to exon, intron, or exon/intron spanning region. The data is from the same species-mixing experiment as the main Figure 1.

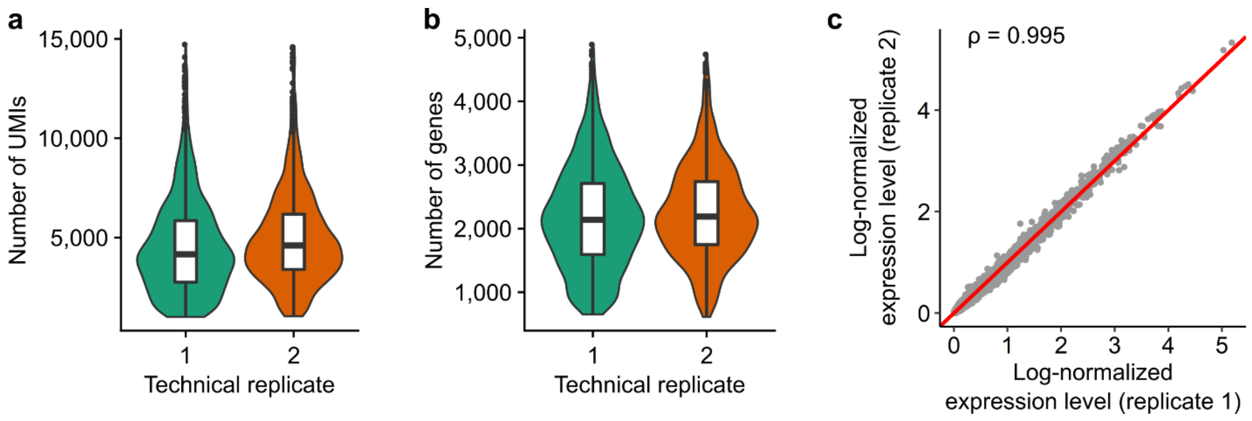

Supplementary Figure 3. Technical replication of FD-seq. (a, b) Violin plots showing the distribution of the number of (a) detected UMIs and (b) detected genes in the two technical replicates. The middle line inside the box indicates the median, the upper and lower edges of the box indicate the first and third quartiles, and the whiskers extend to 1.5× the interquartile range beyond first and third quartiles. **(c)** Scatter plot showing the log-normalized expression level of two technical replicates. Each dot represents the average normalized expression level, and the red line indicates $y = x$. The plot also shows the Pearson's correlation coefficient ρ of the log-normalized expression levels between the two replicates. In (a-c), n=1,483 and 1,338 single cells for replicates 1 and 2, respectively.

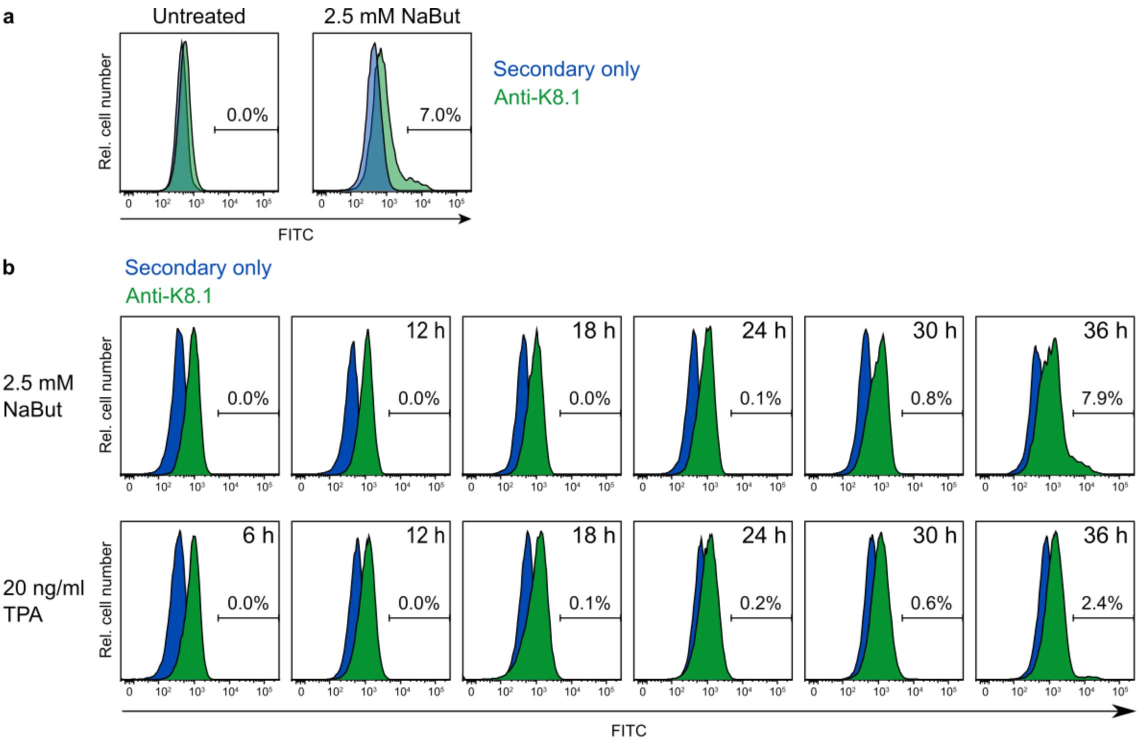

Supplementary Figure 4. Optimization of K8.1 antibody staining and induction of reactivation. (a) Frequency of spontaneous and induced reactivation in BC3 cells. These cells were treated with 2.5 mM of NaBut for 48 hours. **(b)** Time course of reactivation induced by NaBut (top) or TPA (bottom) treatment.

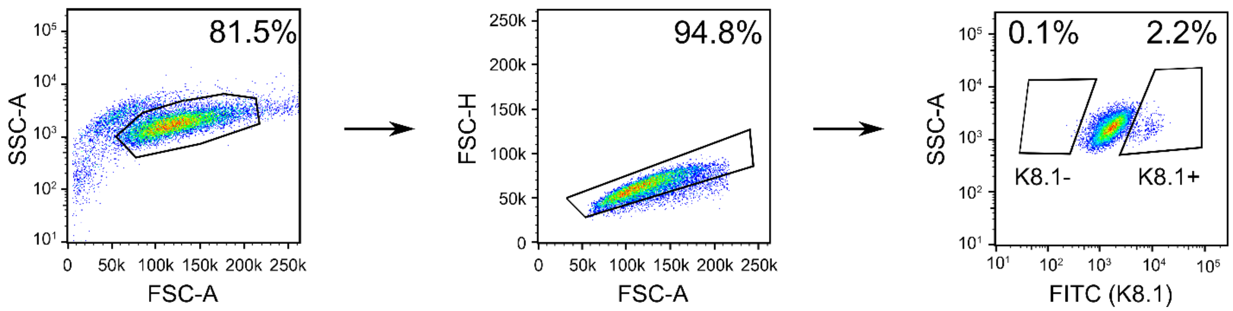

Supplementary Figure 5. Gating strategy for K8.1-positive and K8.1-negative population. This gating strategy was used for FD-seq processing of reactivated and non-reactivated BC3 cells.

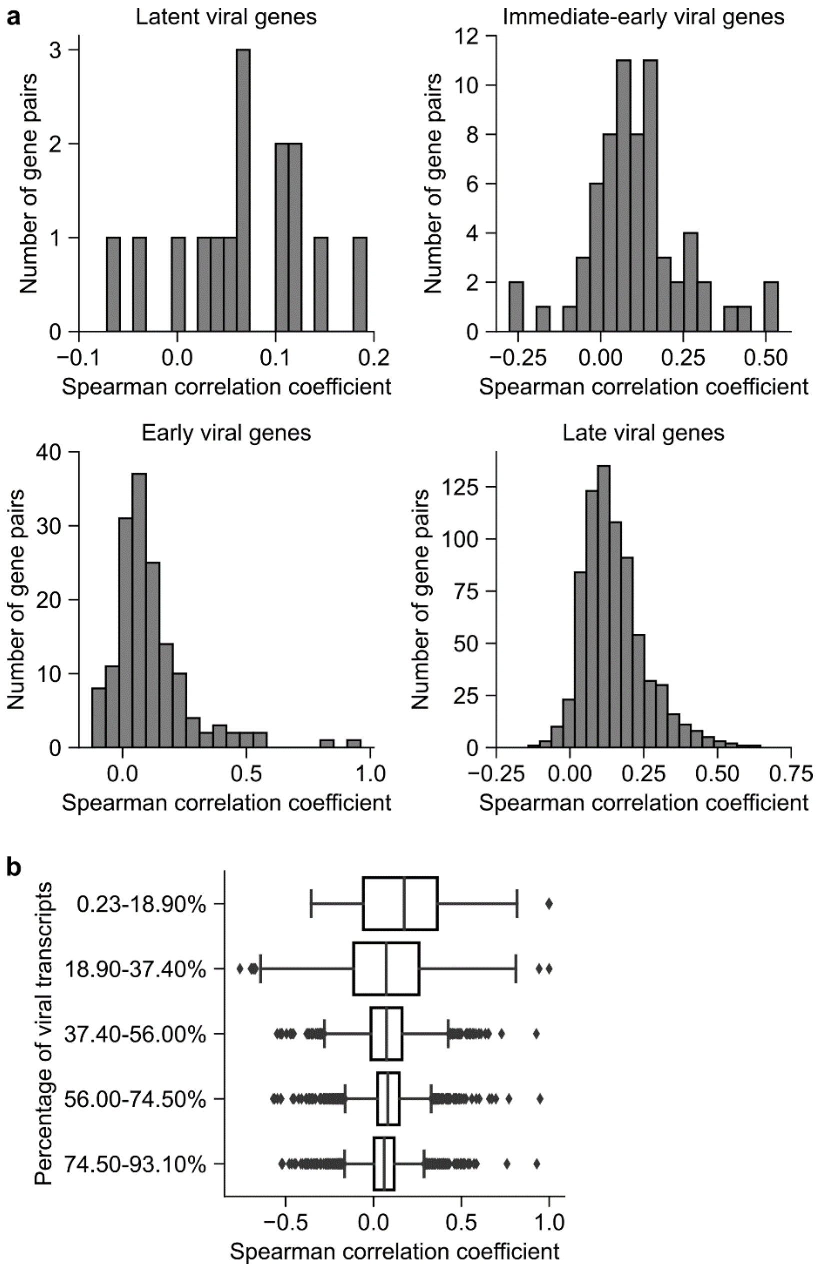

Supplementary Figure 6. Pairwise correlation of viral genes by timing and viral transcript abundance. (a) Histograms showing the pairwise Spearman correlation coefficients of latent, immediate-early, early and late viral genes. **(b)** Box plot showing the pairwise Spearman correlation coefficient between viral transcripts binned by the percentage of viral transcripts. The middle line indicates the median of the data, the box edges indicate the first and third quartile, the whiskers indicate 1.5× the interquartile range beyond the first or third quartile, and the dots indicate the outliers. In (b), the bins have n=1891, 3321, 3570, 3655 and 3655 coefficients, in increasing order of the bins' values.

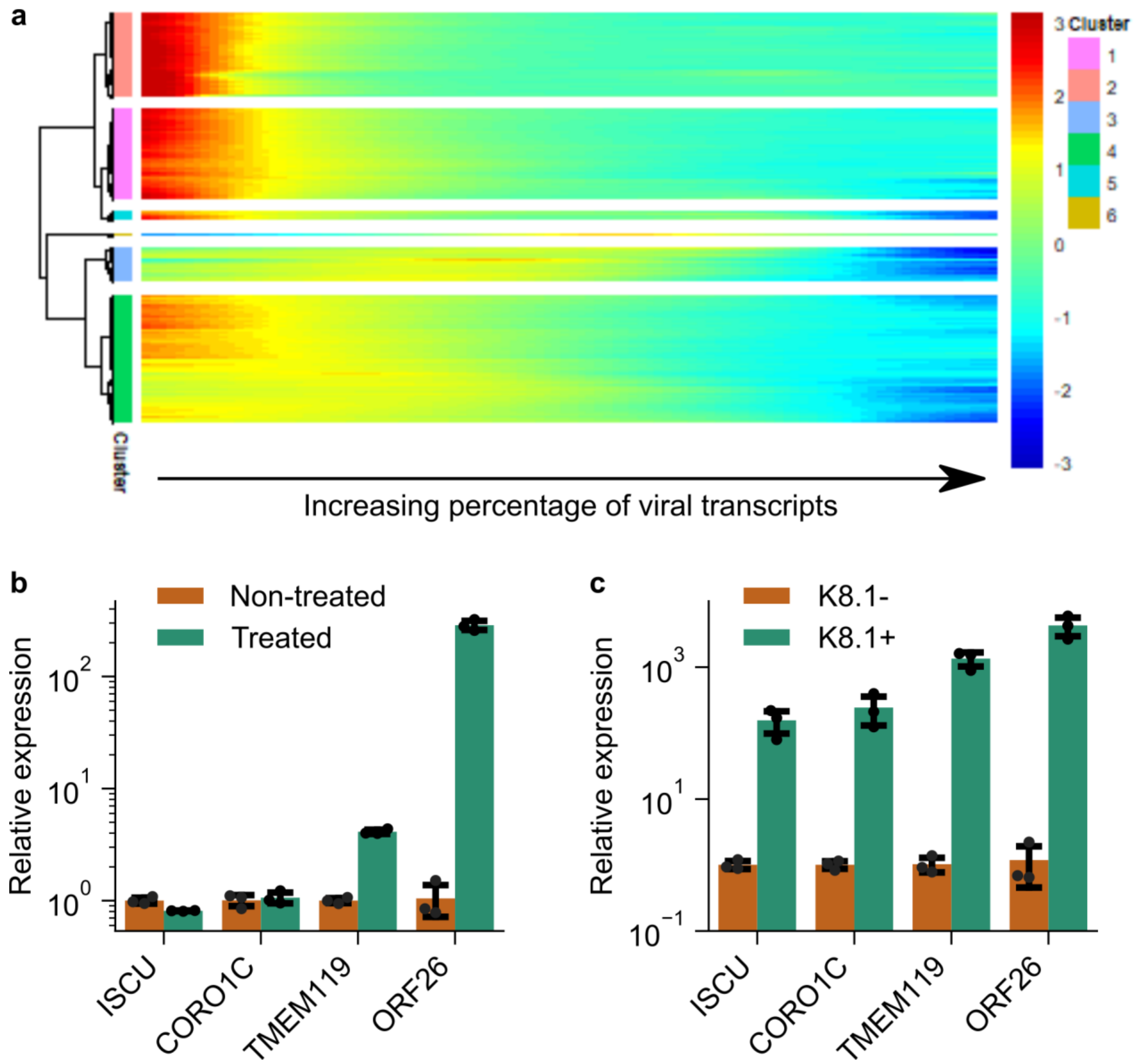

Supplementary Figure 7. Expression of host genes as a function of the abundance of viral transcripts. (a) Heatmap of relative expression level of differentially expressed host genes. Each row shows the relative expression level of a host gene. The genes are clustered based on their expression profile, with only cluster 6 showing a positive correlation with the percentage of viral transcripts. **(b, c)** qPCR validation of the upregulated host genes in reactivated BC3 cells. The cells were treated or not treated with TPA (b), or the treated cells were sorted into K8.1-negative and K8.1-positive subpopulations (c). ORF26 is a viral transcript, and serves as a positive control. (b,c) Data is presented as mean ± standard deviation. n=3 biological replicates.

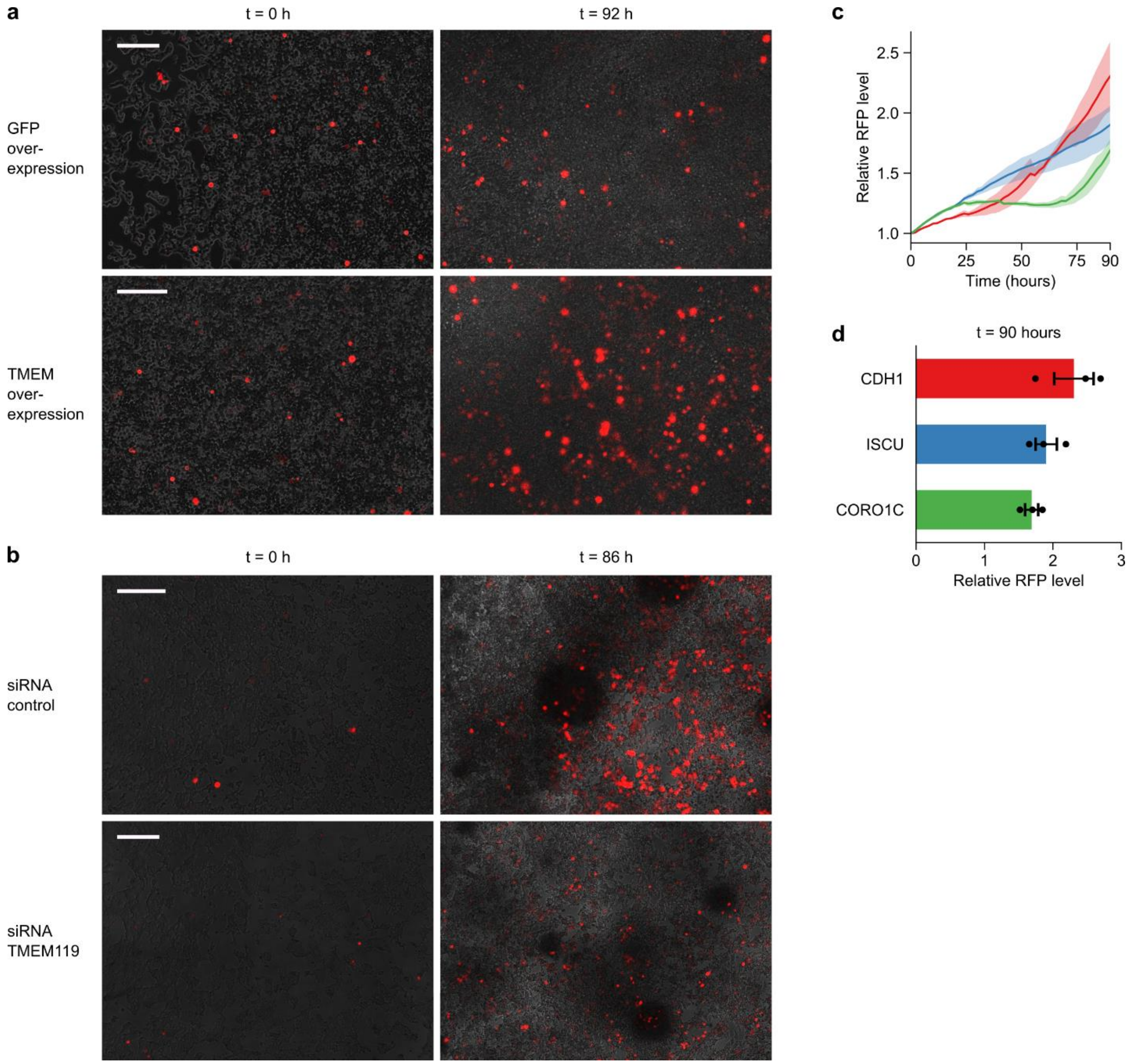

Supplementary Figure 8. Live imaging experiment. (a) Representative images of RFP level, which indicates KSHV reactivation level, in TPA-treated HEK293T.rKSHV219 cells transfected with GFP control (top) or *TMEM119* (bottom) at 0 and 92 hours. **(b)** Representative images of RFP level in HEK293T.rKSHV219 cells transfected with control siRNA (top) or TMEM119 siRNA (bottom) at 0 and 86 hours. The scale bars in (a) and (b) represent approximately 100 µm. **(c)** Time course of RFP level in HEK293T.rKSHV219 cells transfected with *CDH1*, *ISCU* or *CORO1C* genes. **(d)** Endpoint RFP level at t = 90 hours of the time course data in (c). The ribbons in (c) and the error bars in (d) indicate s.e.m. In (a-d), n=3 biological replicates. All live imaging experiment replicates show similar results as the representative images in (a, b).

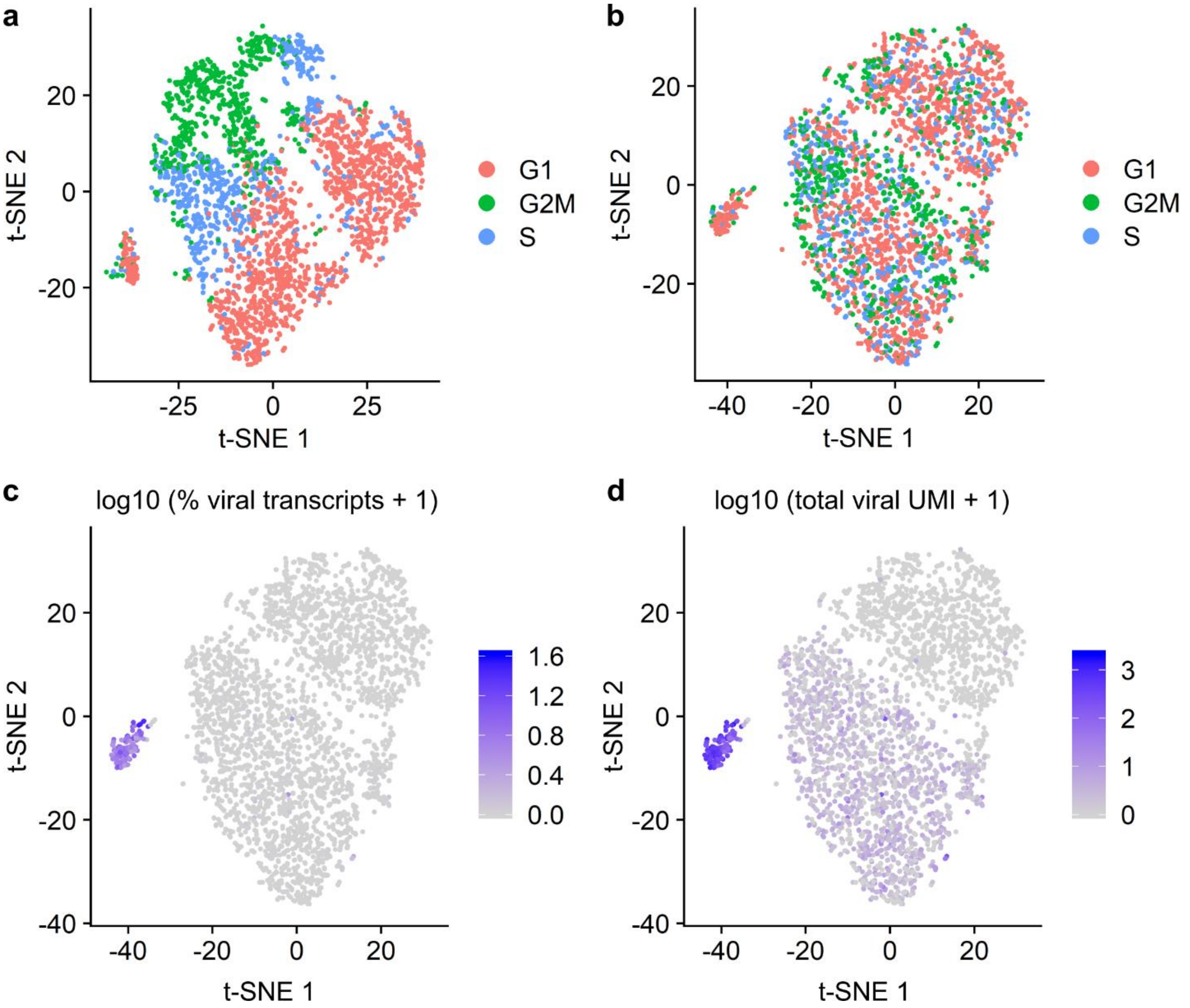

Supplementary Figure 9. Cell cycle effects and the expression of viral transcripts in OC43-infected A549 cells. (a, b) t-SNE plots of cells (a) before and (b) after removing cell cycle effects. The colors indicate the cell cycle scores. **(c, d)** t-SNE plots showing (c) the percentage of viral transcripts and (d) the total UMI of viral transcripts of each single cell.

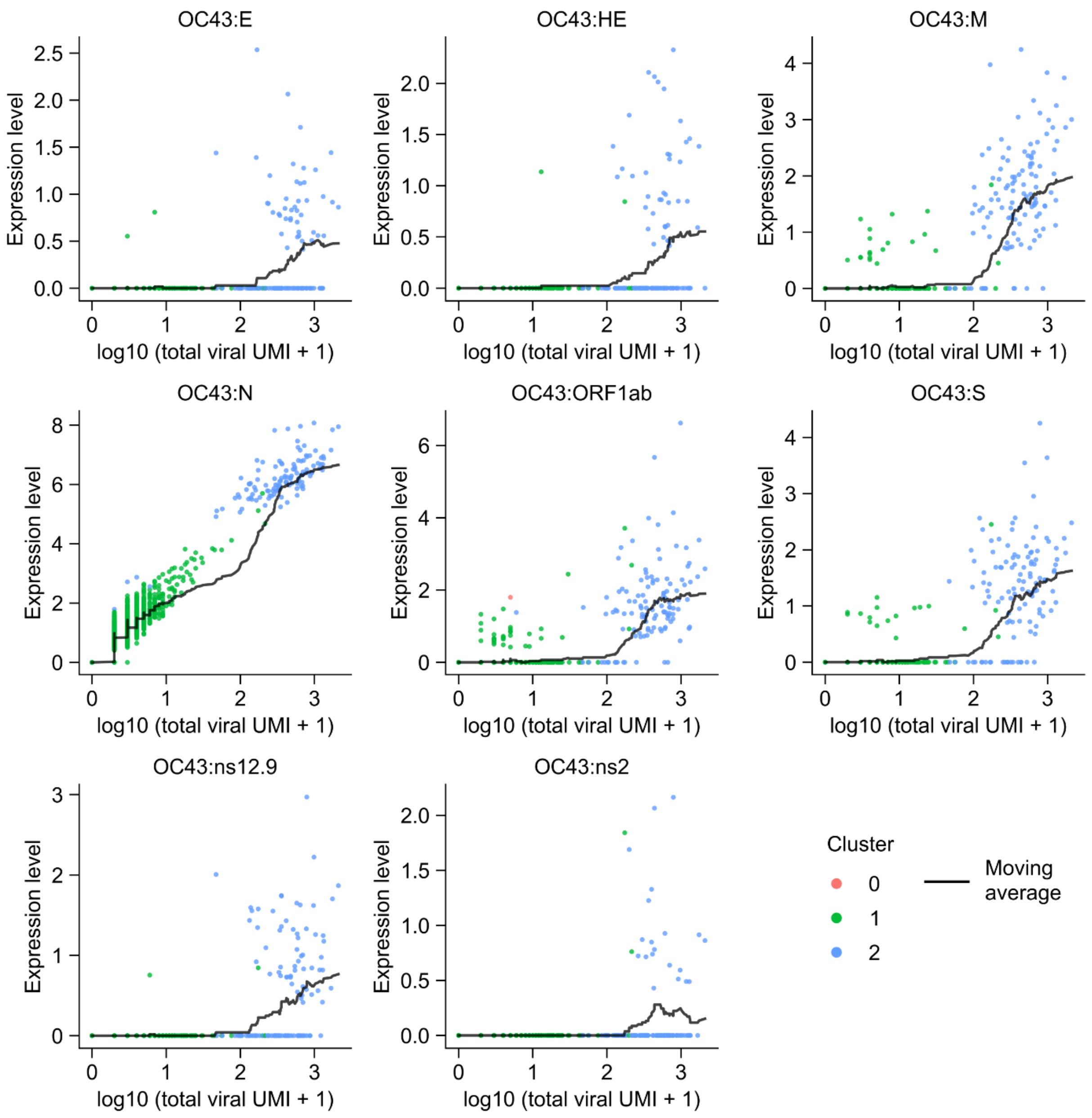

Supplementary Figure 10. Expression profiles of all OC43 viral genes in MOI 1 cells. Scatter plots showing the expression level of each gene against the total abundance of viral genes. The black lines show the 50-cell moving average of the normalized expression level.

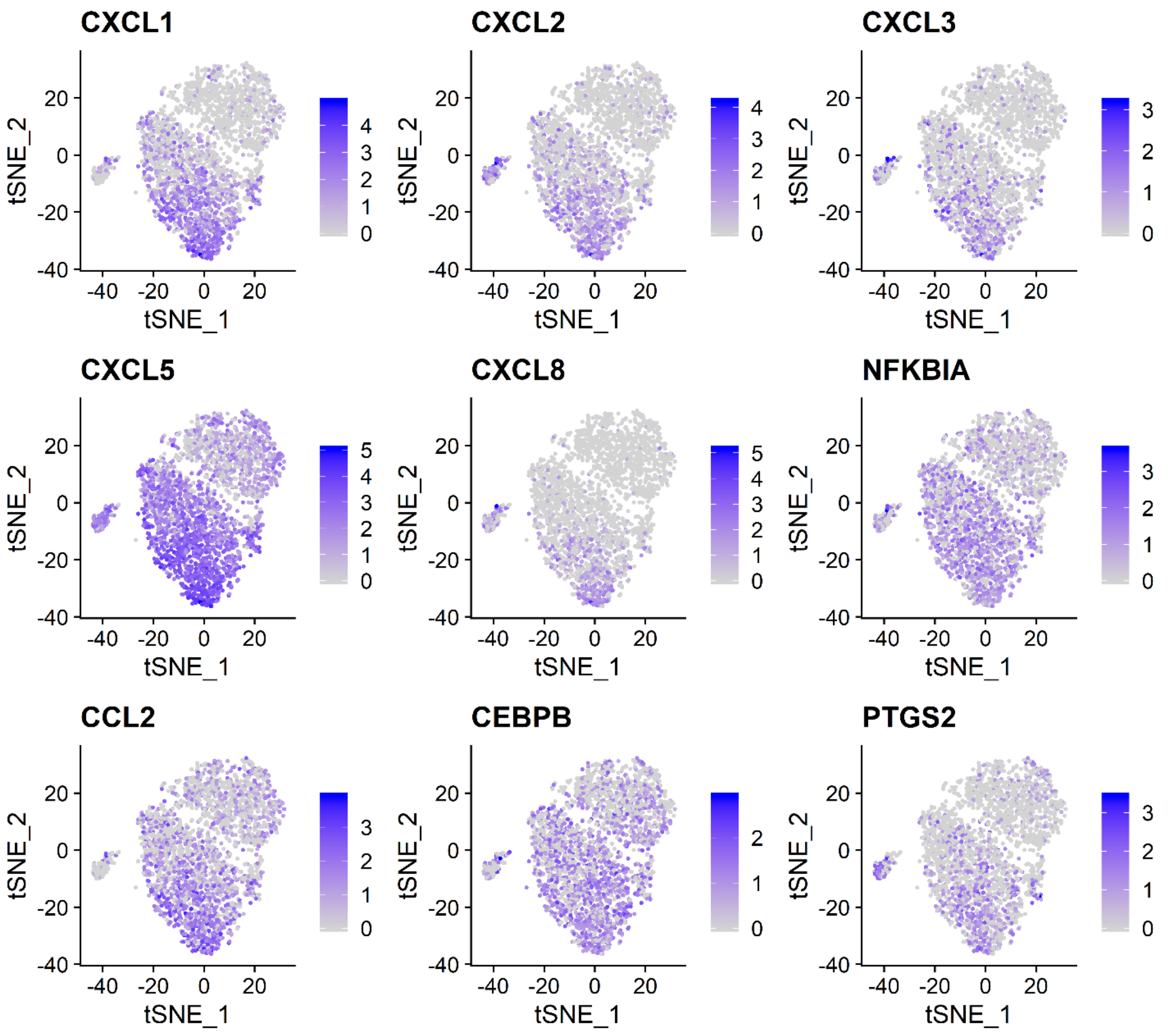

Supplementary Figure 11. Expression level of 9 representative immune-related genes. t-SNE plots showing the log-normalized expression level of the 9 genes.

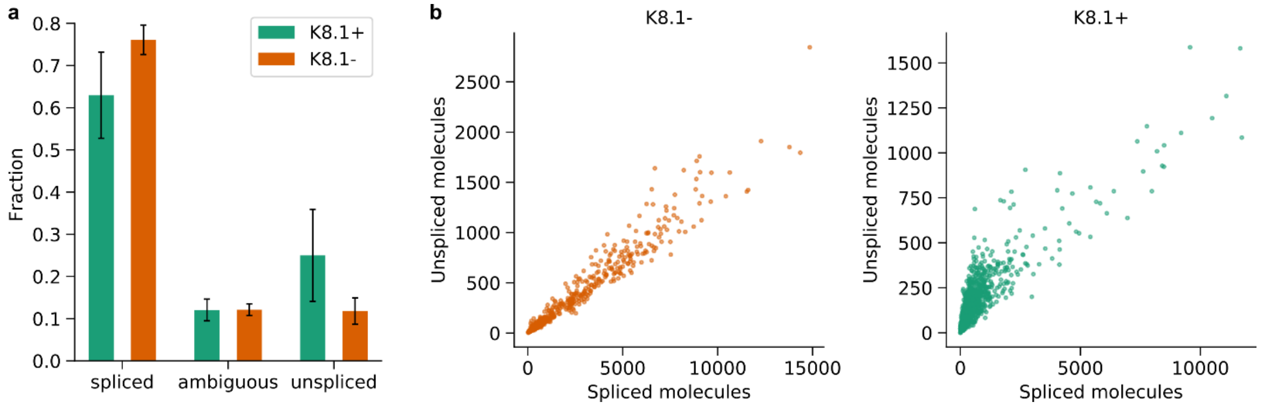

Supplementary Figure 12. Detection of unspliced host mRNAs from fixed BC3 cells. (a) Fractions of spliced, spliced and unassigned mRNA molecules. Data is presented as mean ± standard deviation. **(b)** The number of spliced and unspliced molecules in non-reactivated (left) and reactivated cells (right). In (a, b), n = 1,035 and n = 286 single cells for K8.1+ and K8.1− sample, respectively.

Supplementary Table 1. Primer sequences for FD-seq

Name	Sequence
TSO_RNAhybrid	AAGCAGTGGTATCAACGCAGAGTGAATrGrGrG
TSO_PCR	AAGCAGTGGTATCAACGCAGAGT
P5-TSO_Hybrid	AATGATACGGCGACCACCGAGATCTACACGCCTGTCCGCGGAAGCAGTGGTATCAACGCAGAGT*A*C
Nextera_N701	CAAGCAGAAGACGGCATACGAGATTCGCCTTAGTCTCGTGGGCTCGG
Nextera_N702	CAAGCAGAAGACGGCATACGAGATCTAGTACGGTCTCGTGGGCTCGG
Nextera_N703	CAAGCAGAAGACGGCATACGAGATTTCTGCCTGTCTCGTGGGCTCGG
Nextera_N704	CAAGCAGAAGACGGCATACGAGATGCTCAGGAGTCTCGTGGGCTCGG
Read1CustomSeqB	GCCTGTCCGCGGAAGCAGTGGTATCAACGCAGAGTAC

Supplementary Table 2. Primer sequences for RT-qPCR

Gene	Forward primer	Reverse primer	Probe
GAPDH	GAACATCATCCCTGCCTCTACTG	CAGTGAGCTTCCCGTTCAGC	
ISCU	CTGCACTGCTCCATGCT	CTCATTTCTTCTCTGCCTCTCC	
CORO1C	GTCCACTACCTCAACACATTCA	TGAAGAATCTGGCAATCTCACA	
TMEM119	CCTGGCGTGAAGCAGTATTT	GCACAGGCAGAATGACACTAA	
ORF26	GCTAGCAGTGCTACCCCCATT	GGTCAAATCCGTTGGATTCG	
ORF50	CACAAAAATGGCGCAAGATGA	TGGTAGAGTTGGGCCTTCAGTT	AGAAGCTTCGGCGGTCCTG
ORF57	TGGACATTATGAAGGGCATCCTA	CGGGTTCGGACAATTGCT	TGACGAATCGAGGGACGACGAGA
ORF74 (vGPCR)	GTTCCCCTGATATACTCCTGC	GGACATGAAAGACTGCCTGAG	AGGATGTACGGTCTCTTCCAAAGCC
