# Supplementary material for: High-throughput RNA sequencing of paraformaldehyde-fixed single cells
Source: Nat Commun. 2021 Sep 24;12:5636. doi: 10.1038/s41467-021-25871-2 (PMC8463713; doi:10.1038/s41467-021-25871-2)
Supplement: Supplementary file 2 — Description of Additional Supplementary Files [file 41467_2021_25871_MOESM2_ESM.pdf]

Title: Supplementary Data 1

Description: List of genes that are strongly correlated with the percentage of KSHV viral transcripts in K8.1+ and K8.1− OC43 cells, as measured by FD-seq. This file is related to Figure 4a and Supplementary Figure 7a.

Title: Supplementary Data 2

Description: List of gene markers of each cluster in OC43-infected and mock-infected A549 cells, as measured by FD-seq. This file is related to Figure 5g and Supplementary Figure 11.
