# Supplementary material for: High-throughput RNA sequencing of paraformaldehyde-fixed single cells
Source: Nat Commun. 2021 Sep 24;12:5636. doi: 10.1038/s41467-021-25871-2 (PMC8463713; doi:10.1038/s41467-021-25871-2)
Supplement: Supplementary file 5 — Reporting Summary [file 41467_2021_25871_MOESM5_ESM.pdf]

nature portfolio

Corresponding author(s): Savas Tay

Last updated by author(s): Aug 20, 2021

Reporting Summary

Nature Portfolio wishes to improve the reproducibility of the work that we publish. This form provides structure for consistency and transparency in reporting. For further information on Nature Portfolio policies, see our Editorial Policies and the Editorial Policy Checklist.

Statistics

For all statistical analyses, confirm that the following items are present in the figure legend, table legend, main text, or Methods section.

n/a	Confirmed	
☐	☒	The exact sample size (*n*) for each experimental group/condition, given as a discrete number and unit of measurement
☐	☒	A statement on whether measurements were taken from distinct samples or whether the same sample was measured repeatedly
☐	☒	The statistical test(s) used AND whether they are one- or two-sided<br>*Only common tests should be described solely by name; describe more complex techniques in the Methods section.*
☐	☒	A description of all covariates tested
☐	☒	A description of any assumptions or corrections, such as tests of normality and adjustment for multiple comparisons
☐	☒	A full description of the statistical parameters including central tendency (e.g. means) or other basic estimates (e.g. regression coefficient) AND variation (e.g. standard deviation) or associated estimates of uncertainty (e.g. confidence intervals)
☐	☒	For null hypothesis testing, the test statistic (e.g. *F*, *t*, *r*) with confidence intervals, effect sizes, degrees of freedom and *P* value noted<br>*Give P values as exact values whenever suitable.*
☒	☐	For Bayesian analysis, information on the choice of priors and Markov chain Monte Carlo settings
☒	☐	For hierarchical and complex designs, identification of the appropriate level for tests and full reporting of outcomes
☐	☒	Estimates of effect sizes (e.g. Cohen's *d*, Pearson's *r*), indicating how they were calculated

Our web collection on statistics for biologists contains articles on many of the points above.

Software and code

Policy information about availability of computer code

Data collection	Flow cytometry data was collected using FACS Diva. Sequencing data was obtained from Illumina's BaseSpace platform.
Data analysis	Flow cytometry data was analyzed using FlowJo. Sequencing read alignment was performed using Picard (v2.21.8), Drop-seq tools (v1.13 or v2.3) and STAR Aligner (v2.6.1b). Single-cell count data was analyzed using Monocle 2 or Seurat 3 packages in RStudio. Data visualization was performed using RStudio and Python 3. Splice and unspliced mRNAs were analyzed with dropEst pipeline (v0.8.5) and Python package velocyto (v0.17.17). The code used for sequencing alignment and data analysis is available at https://github.com/tay-lab/FD-seq. g:Profiler (https://biit.cs.ut.ee/gprofiler/gost) was used for KEGG pathway enrichment analysis.

For manuscripts utilizing custom algorithms or software that are central to the research but not yet described in published literature, software must be made available to editors and reviewers. We strongly encourage code deposition in a community repository (e.g. GitHub). See the Nature Portfolio guidelines for submitting code & software for further information.

Data

Policy information about availability of data

All manuscripts must include a data availability statement. This statement should provide the following information, where applicable:

- Accession codes, unique identifiers, or web links for publicly available datasets
- A description of any restrictions on data availability
- For clinical datasets or third party data, please ensure that the statement adheres to our policy

The raw sequencing data and UMI count data generated in this study have been deposited in NBCI’s Gene Expression Omnibus under accession number GSE156988. The human-mouse combed reference genome is available under accession number GSE63269. GQ994935.1 and NC_006213.1 were used as the reference genomes for KSHV and OC43 viral genes, respectively. g:Profiler was used for KEGG pathway enrichment analysis.

Field-specific reporting

Please select the one below that is the best fit for your research. If you are not sure, read the appropriate sections before making your selection.

[x] Life sciences   [ ] Behavioural & social sciences   [ ] Ecological, evolutionary & environmental sciences

For a reference copy of the document with all sections, see nature.com/documents/nr-reporting-summary-flat.pdf

Life sciences study design

All studies must disclose on these points even when the disclosure is negative.

Sample size	The sample size was not pre-calculated. As per standard practices, a sample size of 3 was used to take into account the biological or technical variance. The sample sizes are indicated in the figure captions.
Data exclusions	Low-quality single cells were excluded if their number of detected UMIs is below certain thresholds, and the thresholds are decided based on the UMI distribution of the data set. Low-abundance genes were excluded when they are only detected in fewer than 5 single cells.
Replication	Two technical replicates were performed to assess technical repeatability of FD-seq. For the KSHV reactivation study, we verified the results from scRNA-seq data by performing bulk qPCR and live imaging experiments. All attempts at FD-seq replication were successful. Bulk experiments, such as RNA extraction optimization and RT-qPCR, were performed with three biological replcates.
Randomization	Randomization was not relevant to our study, because we measured all samples from the same experiment at the same time to avoid batch effects.
Blinding	Blinding was not relevant to our study, because group allocation was not performed.

Reporting for specific materials, systems and methods

We require information from authors about some types of materials, experimental systems and methods used in many studies. Here, indicate whether each material, system or method listed is relevant to your study. If you are not sure if a list item applies to your research, read the appropriate section before selecting a response.

Materials & experimental systems

n/a	Involved in the study	
[ ]	[x]	Antibodies
[ ]	[x]	Eukaryotic cell lines
[x]	[ ]	Palaeontology and archaeology
[x]	[ ]	Animals and other organisms
[x]	[ ]	Human research participants
[x]	[ ]	Clinical data
[x]	[ ]	Dual use research of concern

Methods

n/a	Involved in the study	
[x]	[ ]	ChIP-seq
[ ]	[x]	Flow cytometry
[x]	[ ]	MRI-based neuroimaging

Antibodies

Antibodies used	Anti-KSHV K8.1 antibody (sc-65446, Santa Cruz Biotechnology)<br>Alexa Fluor 488-conjugated goat-anti-mouse secondary antibody (A10667, Life Technologies)
Validation	The anti-KSHV K8.1 antibody was validated with flow cytometry, using treated and non-treated BC3 cells to detect expression of the K8.1 viral protein (Supplementary Figure 4).<br>The goat-anti-mouse secondary antibody was validated by the manufacturer: https://www.thermofisher.com/antibody/product/Goat-anti-Mouse-IgG-IgM-IgA-H-L-Secondary-Antibody-Polyclonal/A-10667

Eukaryotic cell lines

Policy information about cell lines

Cell line source(s)	p65-/- 3T3 mouse fibroblast cells were established in Lee et al., Science signaling 2.93 (2009): ra65-ra65. BC3 and A549 cells were purchased from ATCC. A549-H2B-Ruby was generated by Dr Nir Drayman (The University of Chicago, Illinois, USA). HEK293T.rKSHV219 was generated by Dr Michiel van Gent (Cleveland Clinic, Flordia, USA).
Authentication	The cell lines were not authenticated.
Mycoplasma contamination	The cell lines were not tested for mycoplasma contamination.

Commonly misidentified lines (See ICLAC register)	No commonly misidentified cell lines were used.

Flow Cytometry

Plots

Confirm that:

- [x] The axis labels state the marker and fluorochrome used (e.g. CD4-FITC).
- [x] The axis scales are clearly visible. Include numbers along axes only for bottom left plot of group (a 'group' is an analysis of identical markers).
- [x] All plots are contour plots with outliers or pseudocolor plots.
- [x] A numerical value for number of cells or percentage (with statistics) is provided.

Methodology

Sample preparation	Cells were washed once with 1% BSA in PBS, fixed with 4% paraformaldehyde at room temperature for 15 minutes, washed once with 1% BSA/PBS, permeabilized with 0.1% Triton X-100 and 1% BSA in PBS. Blocking was done with 4% BSA/PBS, then staining was performed with anti-K8.1 antibody (1:50 dilution) and Alexa Fluor 488-conjugated goat-anti-mouse secondary antibody (1:4000 dilution) in 1% BSA and 40 U/ml RNase inhibitor, murine (NEB) in PBS. Finally, cells were washed at least twice with 1% BSA and 40 U/ml RNase inhibitor, murine (NEB) in PBS.
Instrument	LSRFortessa flow cytometer andFACSAriaIIIu cell sorter.
Software	Flow cytometry data was analyzed using FlowJo, and the cell sorter was controlled using FACS Diva (BD Biosciences).
Cell population abundance	The sample purity was determined by FD-seq, showing that the sorted populations contain only the reactivated BC3 cells.
Gating strategy	FSC/SSC gating was used to exclude non-cell debris. Reactivated cells are K8.1+. Non-reactivated cells are K8.1-.

- [x] Tick this box to confirm that a figure exemplifying the gating strategy is provided in the Supplementary Information.
